# *Arabidopsis* RETICULON-LIKE4 (RTNLB4) Protein Participates in *Agrobacterium* Infection and VirB2 Peptide-Induced Plant Defense Response

**DOI:** 10.3390/ijms21051722

**Published:** 2020-03-03

**Authors:** Fan-Chen Huang, Hau-Hsuan Hwang

**Affiliations:** 1 Department of Life Sciences, National Chung Hsing University, Taichung 402, Taiwan; seaworld024@hotmail.com; 2 Ph.D. Program in Microbial Genomics, National Chung Hsing University and Academia Sinica, Taichung 402, Taiwan; 3 Innovation and Development Center of Sustainable Agriculture, National Chung Hsing University, Taichung 402, Taiwan

**Keywords:** RTNLB, *Agrobacterium*, plant defense response, VirB2

## Abstract

*Agrobacterium tumefaciens* uses the type IV secretion system, which consists of VirB1-B11 and VirD4 proteins, to deliver effectors into plant cells. The effectors manipulate plant proteins to assist in T-DNA transfer, integration, and expression in plant cells. The *Arabidopsis* reticulon-like (RTNLB) proteins are located in the endoplasmic reticulum and are involved in endomembrane trafficking in plant cells. The *rtnlb4* mutants were recalcitrant to *A. tumefaciens* infection, but overexpression of *RTNLB4* in transgenic plants resulted in hypersusceptibility to *A. tumefaciens* transformation, which suggests the involvement of RTNLB4 in *A. tumefaciens* infection. The expression of defense-related genes, including *FRK1*, *PR1*, *WRKY22*, and *WRKY29*, were less induced in *RTNLB4* overexpression (O/E) transgenic plants after *A. tumefaciens* elf18 peptide treatment. Pretreatment with elf18 peptide decreased *Agrobacterium*-mediated transient expression efficiency more in wild-type seedlings than *RTNLB4* O/E transgenic plants, which suggests that the induced defense responses in *RTNLB4* O/E transgenic plants might be affected after bacterial elicitor treatments. Similarly, *A. tumefaciens* VirB2 peptide pretreatment reduced transient T-DNA expression in wild-type seedlings to a greater extent than in *RTNLB4* O/E transgenic seedlings. Furthermore, the VirB2 peptides induced *FRK1*, *WRKY22*, and *WRKY29* gene expression in wild-type seedlings but not *efr-1* and *bak1* mutants. The induced defense-related gene expression was lower in *RTNLB4* O/E transgenic plants than wild-type seedlings after VirB2 peptide treatment. These data suggest that RTNLB4 may participate in elf18 and VirB2 peptide-induced defense responses and may therefore affect the *A. tumefaciens* infection process.

## 1. Introduction

The typical type IV secretion system (T4SS)-containing phytopathogenic bacterium *Agrobacterium tumefaciens* is well known for its ability to transfer DNA into plant cells. The trans-kingdom DNA transfer ability renders *A. tumefaciens* the most widely used tool to generate transgenic plants [1]. Once the host plants are transformed by *A. tumefaciens,* the infected plant tissues generate tumors, which results in crown gall disease. The host plant wound sites can secrete phenolic compounds, carbohydrates, and hydrogen ions to create acidic environments for repairing cell damage on cell surfaces [2].

The VirA/VirG two-component system in *A. tumefaciens* can detect phenolic compounds, such as acetosyringone (AS), released from plants, and activates downstream *vir* gene expression to help with bacterial infection [3]. One of the Vir proteins, VirD2, binds to the border sequences of the tumor-inducing plasmid (Ti-plasmid), and the T-DNA fragment (called transfer DNA, T-DNA) is processed, generated, and transferred into plant cells. VirD2 covalently binds to the 5’ end of T-DNA and guides T-DNA transfer into plants through a type IV secretion system (T4SS) that contains a transmembrane transporter and a filamentous pilus (T-pilus) comprising VirB1-B11 and VirD4 proteins [4,5].

In addition to VirD2, the VirE2, VirE3, VirD5, and VirF proteins are transferred into plant cells via a T4SS and help with T-DNA transfer and integration into the plant genome [6]. VirE2 protein is a single-stranded DNA binding protein (SSB) that can bind to the single-strand T-DNA and prevent T-DNA degradation by host enzymes [7]. Recent studies demonstrated that VirE2 may enter the plant cells by clathrin-mediated endocytosis, and the pattern of VirE2 migration is consistent with the endoplasmic reticulum (ER) and F-actin network, which suggests that VirE2 may move through the plant cytoskeleton network into the nucleus [8,9,10]. VirD2 and VirE2 interact with several members of the importinα (IMPa) family that mediate nuclear import of NLS-containing proteins in plant cells [11,12]. The host cell nuclear import machinery may be used by *A. tumefaciens* to help with nuclear targeting of T-DNA [2,13].

Upon *A. tumefaciens* infection, VirE2-interacting protein 1 (VIP1) is phosphorylated by mitogen-activated protein kinase 3 (MPK3) and acts as a transcription factor that induces the expression of several stress-responsive genes, such as *PATHOGENESIS-RELATED 1*, and activates stress-signaling transduction cascades to counteract bacterial infection [14,15,16]. In addition, phosphorylation of VIP1 by MPK3 can help with nuclear entry of VIP1 by the plant cell nuclear import system [14,15,16]. However, *A. tumefaciens* may use another effector protein, VirF, to decrease the VIP1-mediated host defense responses by forming a SCF–E3 ligase complex and degrading the VirE2–VIP1 protein complex by the ubiquitin/proteasome system of the host defense mechanism [17,18,19,20]. At the same time, VirF may facilitate the disassembly of the T-complex by the plant ubiquitin–proteasome complex and mediate T-DNA integration into the host chromosome [19,20,21]. Inside plant cells, VIP1-binding F-box protein is a member of the SCF complex and may functionally replace VirF to destabilize the VIP1–VirE2 complex by proteasomal degradation [19]. Thus, *A. tumefaciens* utilizes various bacteria effectors and exploits plant proteins to avoid plant defense responses and secure successful infection.

Plants utilize surface-localized pattern-recognized receptors (PRRs) to detect various microbe- or pathogen-associated molecular patterns (MAMPs/PAMPs) to help plants counteract pathogen infection via PAMP-triggered immunity (PTI) [22,23]. One of the best-characterized PRRs is *Arabidopsis* FLAGELIN-SENSITIVE2 (FLS2), which recognizes and interacts with a conserved N-terminal 22-amino acid peptide of bacteria flagellin, flg22 [24,25]. Upon flg22 perception, FLS2 forms a complex with BRASSINOSTEROID INSENSITIVE 1-associated receptor kinase 1 (BAK1), thus activating the downstream mitogen-activated protein kinases MPK3 and MPK6, which then induces several regulatory factors, *WRKY22*, *WRKY29*, *FLG22-INDUCED RECEPTOR-LIKE KINASE 1* (*FRK1*), other defense-related genes, and reactive oxygen species (ROS) production [26,27,28,29,30,31,32]. Another well-known PRR is the *Arabidopsis* elongation factor-thermo unstable (EF-Tu) receptor (EFR) that activates the plant immune response by recognizing the EF-Tu of *A. tumefaciens* [33]. The N-terminal 18-amino acid peptide of EF-Tu, elf18, has elicitor activity that can induce a rapid oxidative burst and expression of defense-related genes, including *FRK1* and *NDR1/HIN1-like 10* (*NHL10*).

During plant pathogen infections, endomembrane trafficking systems help transport PRRs to the plant cell surface for secretion of defense-related proteins, antimicrobial metabolites, and cell wall components to counteract pathogen invasion [34,35,36]. The functions of vesicle trafficking and integrity of the endomembrane system play important roles in the plant defense response. The reticulon (RTN) proteins are mainly associated with the ER and are involved in neurite growth, endomembrane trafficking, cell division, and apoptosis [37,38]. The plant subfamily of RTN-like proteins (RTNLBs) has 21 members in *Arabidopsis* [39]. Only a few members of RTNLBs have been studied, and five members (AtRTNLB1-4 and 13) are predominantly localized in ER and participate in tubular ER shaping [39,40,41]. A previous study showed that AtRTNLB1, AtRTNLB2, and AtRTNLB4 interacted with *A. tumefaciens* VirB2 and participated in *A. tumefaciens* infection [42]. Furthermore, AtRTNLB1 and -2 mediate the newly synthesized FLS2 translocation from ER to plasma membrane (PM) and affect plant immunity [43].

Furthermore, when AtRTNLB3 or -8 was overexpressed in *Arabidopsis* transgenic plants, the infection rates of *A. tumefaciens* and *Pseudomonas syringae* pv. *tomato* DC3000 (*Pst* DC3000) increased, which suggests that AtRTNLB3 and -8 may participate in plant–pathogen interactions [44]. Moreover, AtRTNLB3 and -6 are involved in the formation of primary plasmodesmata, which interact with the ER system and the virus, typically transporting viral movement proteins via plasmodesmata to further enter the ER membrane for cell–cell spreading. The Potato virus X movement protein accrued within curved ER tubules, which are RTNLB-abundant regions [45,46]. So far, only limited reports demonstrated the RTNLB roles in plant defense responses.

In this study, we showed that when the *RTNLB4* was knocked down or overexpressed, the transformation rates of *A. tumefaciens* were affected. The induced expression of defense-related genes was lower in *RTNLB4* overexpression (O/E) transgenic plants treated with a PAMP, the elf18 peptide, which suggests the involvement of RTNLB4 in plant defense responses. RTNLB4 interacted with *A. tumefaciens* VirB2, a major component of T-pili. Different regions of the processed VirB2 proteins were then used to design five peptides to examine their effects on plant defense gene expression and response. Pretreatment with two VirB2 peptides, S111-T58 and I63-I80, for 6 hrs decreased transient T-DNA expression in wild-type but not *efr-1* and *bak1* mutant seedlings. The two peptides induced relatively higher expression of several defense-related genes, including *FRK1*, *WRKY22*, *WRKY29*, *MPK3*, and *MPK6*, in wild-type plants than in *RTNLB4* O/E transgenic plants. Furthermore, elf18- and VirB2 peptides-mediated *Arabidopsis* seedling growth inhibition and H_2_O_2_ accumulation were reduced in *RTNLB4* O/E transgenic plants. RTNLB4 may have important roles in *A. tumefaciens* elf18 and VirB2 peptide-induced plant defense responses.

## 2. Results

### 2.1. rtnlb4 Mutants Were Recalcitrant to Agrobacterium-Mediated Transformation in Roots and Seedlings

**Figure 1:**
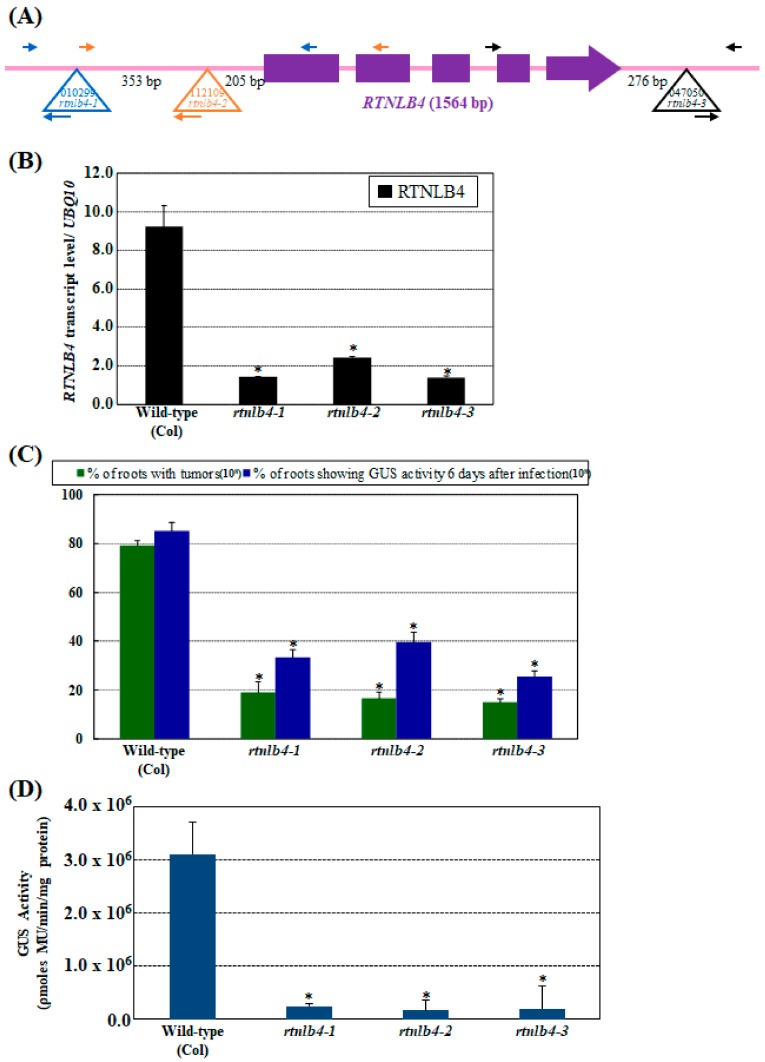
The *Arabidopsis rtnlb4* T-DNA insertion mutants were recalcitrant to *Agrobacterium tumefaciens* transformation. (**A**) Schematic representations of the T-DNA insertion regions around the *Arabidopsis RTNLB4* gene. Purple boxes represent exon regions of the *RTNLB4* gene. The large open triangle represents T-DNA insertion sites in the *RTNLB4* gene. The long and short arrows indicate the locations of primers used in genomic DNA PCR analysis. (**B**) qPCR results of the *RTNLB4* transcript in *rtnlb4-1, rtnlb4-2,* and *rtnlb4-3* mutants. *UBQ10* (polyubiquitin 10) transcript level was an internal control. Data are mean ± SE from at least three PCR reactions of each mutant. (**C**) Transformation efficiencies of three *rtnlb4* mutant lines and wild-type plants. Green bars indicate percentages of root segments forming tumors at 1 month after infection with 10^8^ cfu mL^−1^ tumorigenic *A. tumefaciens* A208 strain. Blue bars show percentages of root segments with GUS activity 6 days after infection with 10^8^ cfu mL^−1^ *A. tumefaciens* At849 strain. Data are mean ± SE from more than 15 plants. At least 80 root segments were examined for each plant. (**D**) Seedlings from three *rtnlb4* mutant lines showed reduced susceptibility to transient transformation. Mutant seedlings were infected with 10^7^ cfu mL^−1^ acetosyringone (AS)-treated *A. tumefaciens* strain for 3 days to determine transient transformation efficiencies. Data are mean ± SE. * *p* < 0.05 compared with the wild-type by pairwise Student’s *t* test.

To determine whether RTNLB4 alone participates in *A. tumefaciens* infection, three *Arabidopsis* mutants with T-DNA insertion in the *RTNLB4* gene (Figure 1A) were obtained and used to test their susceptibility with root- and seedling-based *A. tumefaciens* transformation assays. In the *rtnlb4-1*, *rtnlb4-2*, and *rtnlb4-3* single mutants, T-DNA insertions were inserted in the promoter, 5′, and 3′ untranslated region (UTR) of the *RTNLB4* gene, respectively (Figure 1A). On quantitative real-time PCR (qPCR), the *RTNLB4* mRNA level was decreased to 13.3% to 23.3% of the wild-type level in the three *rtnlb4* mutants (Figure 1B). Stable *A. tumefaciens*-mediated root transformation assays demonstrated that tumor formation rates decreased more than 4-fold in the three *rtnlb4* mutants as compared with wild-type plants (Figure 1C). The transient transformation rates of three *rtnlb4* mutants reduced more than 53.5% and more than 95.6% compared to wild-type plants when root and seedling tissues, respectively, were used as plant materials (Figure 1C,D). These data indicate that the T-DNA insertions in the three *rtnlb4* mutants result in lower *RTNLB4* expression and a concomitant decrease in *A. tumefaciens*-mediated transformation. Furthermore, the decreased level of transient transformation rate was greater in seedlings than in root tissues of *rtnlb4* mutants, which suggests that successful *A. tumefaciens* transformation in seedlings might be more dependent on *RTNLB4* than in roots.

### 2.2. Overexpression of RTNLB4 in Transgenic Plants Enhanced A. tumefaciens Infection Rates in Roots and Seedlings

**Figure 2:**
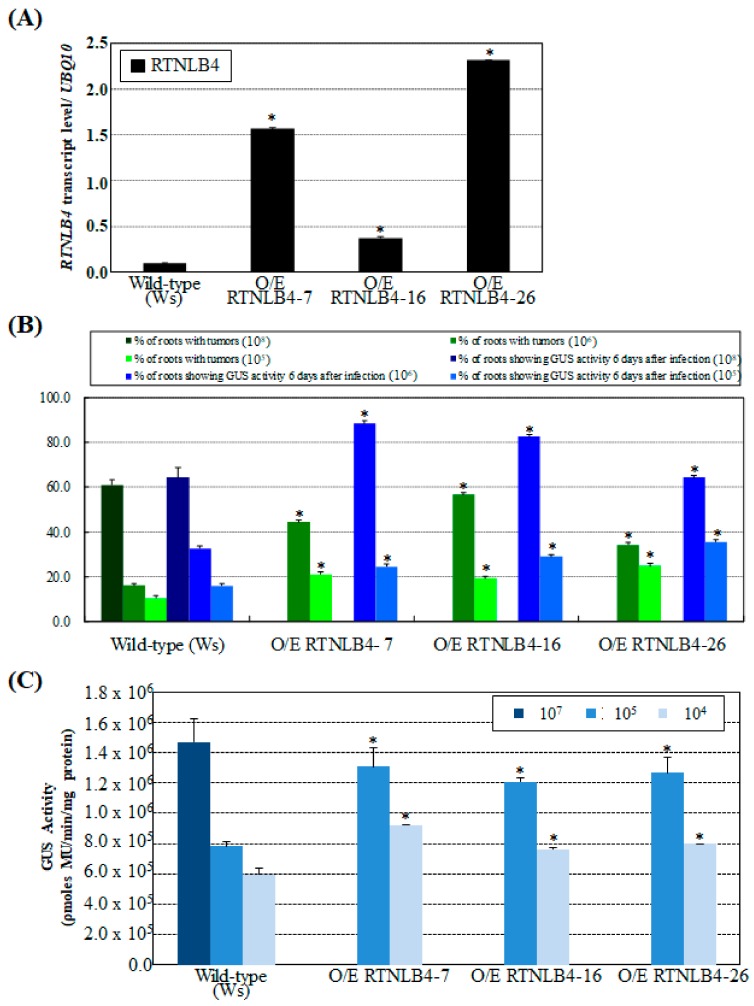
*RTNLB4* overexpression (O/E) transgenic plants were more susceptible to *A. tumefaciens* infections. (**A**) qPCR analysis of *RTNLB4* transcript levels in *RTNLB4* O/E and wild-type plants. The *UBQ10* (polyubiquitin 10) transcript level was an internal control. Data are mean ± SE. (**B**) Stable and transient transformation efficiencies of *RTNLB4* O/E and wild-type plants. Green bars show the percentage of root segments with tumors after infection with 10^8^, 10^6^, or 10^5^ cfu mL^−1^ *A. tumefaciens* A208. Blue bars represent the percentage of root segments with GUS activity after infection with 10^8^, 10^6^, or 10^5^ cfu mL^−1^ *A. tumefaciens* At849 strain. *A. tumefaciens* at 10^8^ cfu mL^−1^ was used to infect wild-type roots as a positive control to indicate successful transformation. Data are mean ± SE from more than 15 plants. At least 80 root segments were examined for each plant. (**C**) Transient transformation efficiency in seedlings of *RTNLB4* O/E and wild-type plants. Seedlings of O/E plants were infected with 10^5^ or 10^4^ cfu mL^−1^ AS-induced *A. tumefaciens* strain. Wild-type seedlings were infected with 10^7^ cfu mL^−1^ *A. tumefaciens* strain as a positive control. Data are mean ± SE. * *p* < 0.05 compared with the wild-type by pairwise Student’s *t* test.

Because seedlings from three *rtnlb4* mutant lines were resistant to *A. tumefaciens* infection, we next determined whether overexpression of *RTNLB4* in plants could affect *A. tumefaciens* infection rates. We generated transgenic *Arabidopsis* plants that overexpressed *RTNLB4* or T7-tagged-*RTNLB4* by using a double CaMV 35S promoter and performed *A. tumefaciens* infection assays. *RTNLB4* mRNA levels were significantly higher in *RTNLB4* overexpression (O/E) than wild-type plants (Figure 2A). Protein gel blot analysis with anti-T7-tag antibody showed accumulation of T7-tagged-RTNLB4 recombinant proteins in T7-tagged-*RTNLB4* O/E transgenic plants (Figure S1A). Relatively lower concentrations of *A. tumefaciens,* 10^6^ and 10^5^ cfu mL^−1^ were used to infect root tissues of *RTNLB4* O/E transgenic plants. Both stable and transient transformation rates of *RTNLB4* and T7-tagged-*RTNLB4* O/E plants were enhanced more than 1.5-fold as compared with wild-type plants (Figure 2B and Figure S1B). Similarly, *RTNLB4* O/E seedlings showed more than 1.5-fold increased GUS activity as compared with wild-type seedlings (Figure 2C) when 10^5^ and 10^4^ cfu mL^−1^ bacteria concentrations were used to infect seedlings. These data indicate that overexpression of *RTNLB4* may increase plant susceptibility to *A. tumefaciens* infection, and the presence of the T7 tag sequence in the N-terminal region of RTNLB4 proteins may not affect the RTNLB4 protein functions during *A. tumefaciens* infection. The data suggest the RTNLB4 protein may participate in steps prior to T-DNA integrations during *A. tumefaciens* infection.

### 2.3. Induced Expression of Defense-Related Genes Was Affected in both RTNLB4 O/E Transgenic Plants and rtnlb4 Mutants after elf18 Treatment

**Figure 3:**
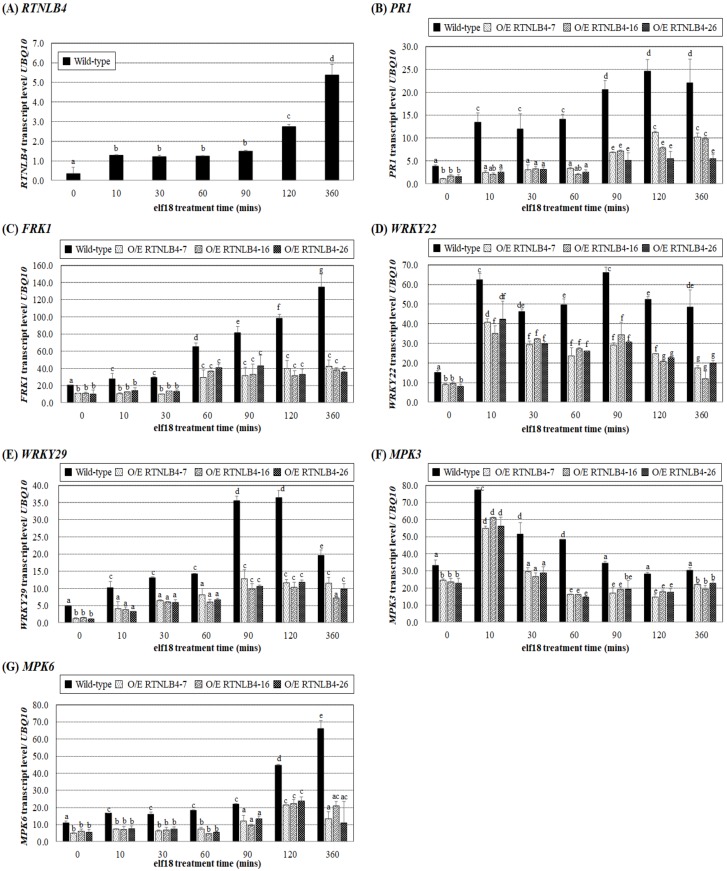
Induction of defense-related genes by elf18 was significantly impaired in *RTNLB4* O/E transgenic plants. Gene expression of *RTNLB4* (**A**), *PR1* (**B**), *FRK1* (**C**), *WRKY22* (**D**), *WRKY29* (**E**), *MPK3* (**F**), and *MPK6* (**G**) in seedlings of *RTNLB4* O/E transgenic and wild-type treated with 10 µM elf18 for 0, 10, 30, 60, 90, 120, and 360 min measured by qPCR analysis. The *UBQ10* (polyubiquitin 10) transcript level was an internal control. Data are mean ± SE from at least three independent biological experiments. Data were analyzed by Duncan test, and means with the same letter (a–g) were not significantly different (*p* < 0.05).

To understand how *RTNLB4* participates in *A. tumefaciens* infection, we first determined whether *RTNLB4* gene expression could be induced by the elf18 peptide of an *A. tumefaciens*-derived PAMP, EF-Tu. We treated *Arabidopsis* seedlings with 10 uM elf18 for up to 6 hrs. *RTNLB4* mRNA level was increased more than 4-fold after 10 min peptide treatment as compared with the 0 min treatment control (Figure 3A). After 120 min of elf18 treatments, the *RTNLB4* mRNA level was significantly 8-fold increased and continuously increased after 360 min peptide treatment (Figure 3A), which suggests the *RTNLB4* might participate in *A. tumefaciens* PAMP-induced plant responses.

Next, we treated wild-type (ecotype: Ws) and *RTNLB4* O/E transgenic plants with elf18 peptide for 6 hrs and examined the expression of several defense-related genes. The mRNA levels of *PR1, FRK1, WRKY22, WRKY29, MPK3*, and *MPK6* were lower in *RTNLB4* O/E transgenic than wild-type plants without peptide treatment (0 min treatment control) (Figure 3B–G). With increased elf18 peptide treatment time, the mRNA levels of *PR1, FRK1, WRKY29,* and *MPK6* were all significantly increased in wild-type plants (ecotype: Ws) (Figure 3B,C,E,G), so the EF-Tu-derived peptide elf18 induced the expression of plant defense genes. *PR1* and *WRKY29* mRNA levels were induced more than 2-fold after 10 min peptide treatment and peaked after 90 min treatment (Figure 3B,E). Similarly, *FRK1* and *MPK6* mRNA levels increased after 10 min treatment and peaked after 6 hr peptide treatment (Figure 3C,G). *WRKY22* and *MPK3* mRNA levels peaked after 10 min peptide treatment (Figure 3D,F). In the three *RTNLB4* O/E transgenic plants, all tested genes were induced after elf18 treatment, but the induced levels were significantly lower than in wild-type plants at the same time (Figure 3B–G). Collectively, these data demonstrate that *RTNLB4* O/E transgenic plants had lower levels of elf18-induced defense-related genes than did wild-type plants. These data also suggest that lower defense-related gene expression in these transgenic plants might help *RTNLB4* O/E transgenic plants hyper-susceptible to *A. tumefaciens* infection.

The wild-type (ecotype: Columbia) and seedlings from three rtnlb4 mutant lines were also treated with elf18 peptide, and the expression of defense-related genes was determined at different times. Without elf18 peptide treatment (0 min treatment control), the mRNA levels of PR1, FRK1, WRKY22, WRKY29, MPK3, and MPK6 were similar to the wild-type plants (Figure S2A–F). Levels of all the examined genes were induced in wild-type plants (ecotype: Columbia) after elf18 peptide treatment (Figure S2A–F). PR1, FRK1, WRKY22, WRKY29, and MPK6 mRNA levels in wild-type plants were steadily increased after 10 min peptide treatment and peaked at later times of peptide treatments (Figure S2A–D,F). In the wild type, MPK3 mRNA level peaked right after 10 min elf18 treatment and decreased at later times of peptide treatments (Figure S2E). PR1, FRK1, WRKY29, and MPK6 mRNA levels were less induced in the three rtnlb4 mutants than in wild-type plants at the same time, and the induced levels of four genes in wild-type plants were relatively higher at later time points than at earlier time points (Figure S2A,B,D,F). In the three rtnlb4 mutants, WRKY22 and MPK3 were induced only after 60 and 10 min elf18 treatment, respectively (Figure S2C,E). These data indicate that the mRNA levels of elf18-induced defense-related genes were also reduced in the three rtnlb4 mutants.

As shown in Figure 1C,D, the rtnlb4 mutants were less susceptible to A. tumefaciens infections. To our surprise, in the rtnlb4 mutants without elf18 peptide treatments, the basal levels of defense-related genes were not significantly higher than wild-type plants. These data might suggest that defense-related gene expression levels in mutants might not be the only cause for the recalcitrance phenotype in the rtnlb4 mutants. Because the rtnlb4 mutants showed lower levels of elf18-induced defense-related gene expression, we next examined the effects of elf18 peptide pretreatments on A. tumefaciens-mediated transformation in rtnlb4 mutants.

### 2.4. Agrobacterium-Mediated Transient Expression Efficiency Is Decreased in Wild-Type Plants and in RTNLB4 O/E Transgenic Plants to a Lesser Extent after elf18 Peptide Pretreatment

**Figure 4:**
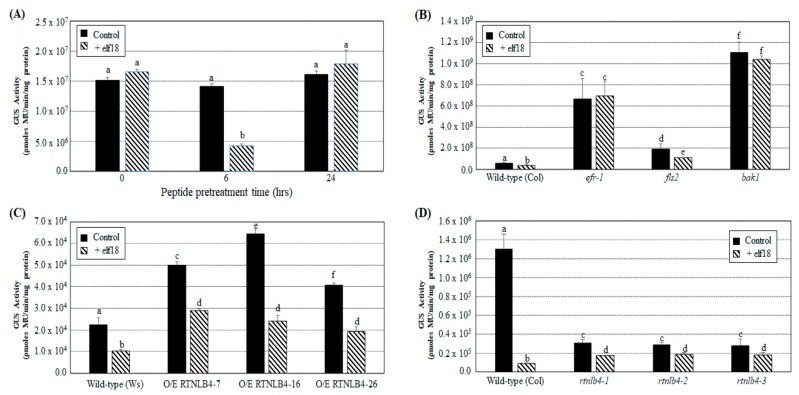
Pretreatment with elf18 peptide reduced transient transformation efficiency in wild-type, *RTNLB4* O/E transgenic, and three *rtnlb4* mutant seedlings. (**A**) Transient transformation efficiencies of wild-type seedlings pretreated with 10 µM elf18 peptide for 0, 6, or 24 hr before infection with *A. tumefaciens*. Transient transformation rates of wild-type, *efr-1*, *fls2*, and *bak1* mutants (**B**), *RTNLB4* O/E transgenic plants (**C**), and three *rtnlb4* mutant plants (**D**) pretreated with 10 µM elf18 peptide for 6 h before infection with *A. tumefaciens.* Distilled H_2_O (dH_2_O) was used as the mock control in seedling transient transformation assays. Data are mean ± SE from at least three independent transformation assays. Data were analyzed by Duncan tests and means with different letters were significantly different (*p* < 0.05).

Because the *A. tumefaciens*-derived PAMP elf18 peptide induced the expression of plant defense-related genes (Figure 3B,G and Figure S2A–F), we further tested the effect of elf18-induced plant defense on *A. tumefaciens*-mediated transient expression in *Arabidopsis* seedlings. Wild-type plant seedlings were first treated with 10 μM elf18 peptide for 0, 6, or 24 h before *A. tumefaciens*-mediated transient transformation assays. The elf18 peptide was dissolved in distilled H_2_O (dH_2_O), and dH_2_O was then used as the mock control in seedling transient transformation assays. Only pretreatment with elf18 for 6 h significantly decreased GUS activity in wild-type seedlings after *A. tumefaciens* transformation as compared with the mock control under the same pretreatment time (Figure 4A). These data correlate well with the levels of several defense-related genes induced after 6 h of elf18 treatments. They also suggest that defense responses activated by elf18 may affect subsequent plant transformation by *A. tumefaciens*. We also treated the EF-Tu receptor mutant *efr-1*, the flagellin receptor mutant *fls2*, and *bak1* mutants with the elf18 peptide for 6 h before *A. tumefaciens-*mediated transformation. Without elf18 pretreatment, GUS activity was highest in the *bak1* mutant, followed by *efr-1*, *fls2*, and finally wild-type (ecotype: Col) plants (Figure 4B). These results are consistent with previous published data [33,47] showing that defects in the PAMP-induced defense response may increase plant susceptibility to *A. tumefaciens* infection. In addition, only the *fls2* mutant but not the *efr-1* and *bak1* mutants showed decreased GUS activity with 6 hr pretreatment with elf18 as compared with the mock control (Figure 4B). These results confirm previous observations that elf18 activated plant defense responses via an EFR-mediated pathway and restricted *A. tumefaciens* infection [33,47].

Next, wild-type (ecotype: Col or Ws), *RTNLB4* O/E transgenic and *rtnlb4* mutant seedlings were pretreated with elf18 peptide for 6 hrs and then infected with *A. tumefaciens* to determine transient T-DNA expression efficiencies. GUS activities in the wild-type (ecotype: Ws) and *RTNLB4* O/E transgenic seedlings were decreased more than 40% after elf18 pretreatment as compared with the mock control of the same type of plants (Figure 4C). With elf18 pretreatment, GUS activity in *RTNLB4* O/E plants was almost 2-fold higher than in wild-type plants under the same peptide pretreatments (Figure 4C). These data suggest that the lower expression of elf18-induced defense-related genes in *RTNLB4* O/E transgenic plants may help transgenic plants increase their susceptibilities to *A. tumefaciens* transformation.

Without elf18 pretreatment, GUS activity was relatively lower in *rtnlb4* mutants than wild-type plants, which was consistent with data shown in Figure 1D. However, pretreatment with elf18 decreased GUS activity 14.4-fold in wild-type seedlings (ecotype: Col) but only 1.5- to 1.8-fold in *rtnlb4* mutants (Figure 4D). Of note, after elf18 peptide pretreatment, GUS activity became almost 2-fold higher in the *rtnlb4* mutants than in wild-type plants under the same peptide pretreatments (Figure 4D). These data show that after elf18 peptide pretreatments, *rtnlb4* mutants showed relatively higher transient transformation rates than wild-type plants. These data suggest that the relatively lower reduction in GUS activity in *rtnlb4* mutants might be due to less expression of elf18-induced defense-related genes in *rtnlb4* mutants as compared with wild-type plants. These data might also indicate that abnormal high and low levels of the *RTNLB4* mRNA expression in transgenic plants and mutants influence the expression of elf18-induced defense-related genes and therefore affect *A. tumefaciens* transformation efficiency after peptide pretreatments.

### 2.5. VirB2 Peptide Pretreatment Affected Transient T-DNA Expression in Wild-Type, RTNLB4 O/E Transgenic, and rtnlb4 Mutant Plants

**Figure 5:**
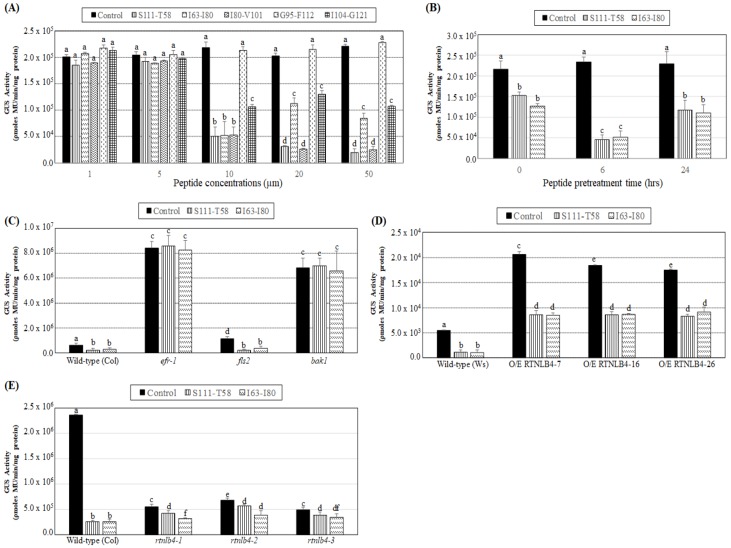
Transient transformation rates of wild-type, *RTNLB4* O/E transgenic and three *rtnlb4* mutant seedlings were decreased with VirB2 peptide pretreatments. (**A**) Transient transformation rates of wild-type seedlings pretreated with 1, 5, 10, 20, or 50 µM of five VirB2 peptides, S111-T58, I63-I80, I80-V101, G95-F112, or I104-G121, for 6 hr before infection with *A. tumefaciens*. (**B**) Transient transformation efficiency of wild-type seedlings pretreated with 10 µM of two VirB2 peptides, S111-T58 or I63-I80, at 0, 6, or 24 hr before infection with *A. tumefaciens*. *Agrobacterium*-mediated transient transformation rates of wild-type, *efr-1*, *fls2*, *bak1* mutants (**C**), *RTNLB4* O/E transgenic plants (**D**), and three *rtnlb4* mutant plants (**E**) pretreated with 10 µM of the two VirB2 peptides for 6 hrs before infection with *A. tumefaciens.* DMSO solution was used as the mock control in seedling transient transformation assays. Data are mean ± SE from at least three independent transformation assays. Data were analyzed by Duncan tests and means with different letters were significantly different (*p* < 0.05).

Because the *A. tumefaciens* T-pilus mainly consists of the VirB2 protein and several RTNLB proteins showed interactions with VirB2 proteins [42,44], we examined whether the VirB2 peptide could induce plant defense responses and affect *A. tumefaciens* transformation. In *A. tumefaciens*, the signal peptide of VirB2 is cleaved, followed by the first and last amino acids being linked to form an unusual cyclic peptide [48,49,50]. Five peptides of the VirB2 protein with 18-22 amino acids (Table 1) were used to pretreat wild-type seedlings, and we examined their effects on *A. tumefaciens* transient transformation efficiency. VirB2 peptides were dissolved in DMSO, and the DMSO solution was used as the mock control in transformation assays. Five VirB2 peptides with five different peptide concentrations, 1, 5, 10, 20, or 50 µM, were used to pretreat seedlings for 6 hr. GUS activities were similar with 1 and 5 µM VirB2 peptide used to pretreat wild-type seedlings as compared with the mock control (Figure 5A). With VirB2 peptide concentration increased to 10 µM, three VirB2 peptides, S111-T58, I63-I80, and I80-V101, showed the highest reduction of GUS activity, followed by I104-G121, and G95-F112 peptide did not show a significant reduction of GUS activity as compared with the mock control (Figure 5A). With VirB2, peptide concentration increased to 20 µM and 50 µM, four VirB2 peptides, S111-T58, I63-I80, I80-V101, and I104-G121, decreased GUS activity 1.5- to 10-fold in wild-type seedlings as compared with the mock control under the same peptide concentration treatments (Figure 5A). Among the five tested VirB2 peptides, S111-T58 and I63-I80 had better ability to reduce *A. tumefaciens* transformation and relatively lower hydrophobicity (Figure 5A and Table 1). S111-T58 and I63-I80 at 10 µM were further tested with different pretreatment times. Wild-type seedlings pretreated with the VirB2 peptides for 6 h conferred more than 4-fold reduction of GUS activity (Figure 5B), whereas pretreatments for 0 and 24 h conferred only 1.5-fold reduction of GUS activity as compared with the mock control under the same pretreatment times. These data were consistent with results obtained with the elf18 peptide (Figure 4A).

Because the VirB2 peptides S111-T58 and I63-I80 restricted *A. tumefaciens* infection, we pretreated the *efr-1, fls2*, and *bak1* mutants with the VirB2 peptides for 6 hrs to decipher further the VirB2 peptide-induced plant defense responses. GUS activities of the wild type and *fls2* mutant were both significantly decreased as compared with the mock control of the same kind of plants after pretreatment with the two VirB2 peptides (Figure 5C). GUS activity of the *efr-1* and *bak1* mutants showed no difference from the mock control after VirB2 peptide pretreatment (Figure 5C), which suggests that the VirB2 peptides S111-T58 and I63-I80 may induce plant defense responses mainly through EFR and BAK1 proteins, which is similar to the *A. tumefaciens* PAMP elf18-induced plant defense responses.

Both RTNLB4 O/E transgenic and rtnlb4 mutant plants were defective in elf18-induced plant defense responses. Therefore, we pretreated RTNLB4 O/E transgenic and rtnlb4 mutant plants with the two VirB2 peptides S111-T58 and I63-I80 for 6 hr and observed VirB2 peptide effects on A. tumefaciens transformation of these plants. GUS activity in the wild type was reduced more than 4-fold after pretreatment with the two VirB2 peptides, whereas GUS activity in three RTNLB4 O/E transgenic plants was reduced only 2-fold as compared with the mock control of the same type of plants (Figure 5D). Similarly, the reduction in GUS activity in three rtnlb4 mutants was less than in wild-type seedlings after pretreatment with the two VirB2 peptides (Figure 5E). Collectively, these data showed that after VirB2 peptide pretreatments, both RTNLB4 O/E transgenic plants and rtnlb4 mutants had higher transient transformation rates than wild-type plants. These data also suggest that when the RTNLB4 mRNA level is affected, the inhibition level of A. tumefaciens-mediated transformation by VirB2 peptide pretreatments was less than wild-type plants.

### 2.6. Levels of Defense-Related Genes in RTNLB4 O/E Transgenic and rtnlb4 Mutant Plants Were Less Induced after VirB2 Peptide Treatment

Pretreating *Arabidopsis* plants with the two VirB2 peptides S111-T58 and I63-I80 impeded *A. tumefaciens* infection, so we determined whether the VirB2 peptides may induce the expression of plant defense-related genes. Because the major component of the *A. tumefaciens* T-pilus is VirB2, we first examined whether T-pili may affect the expression of plant defense-related genes. The mRNA levels of *FRK1* and *WRKY22* increased in wild-type seedlings after 60 and 30 min T-pili treatments, respectively (Figure S3A,B). In addition to the general plant defense-related genes, another elf18-induced gene, *CYTOCHROME P450, FAMILY 81* (*CYP81F2*), encoding an indole glucosinolate biosynthetic protein [55,56], was induced by T-pili after 60 min treatment (Figure S3C). The expression levels of an elf18- and flg22-induced gene At2g17740, encoding a Divergent C1 (DC1)-domain containing protein [57], was also increased after 60 min T-pili treatment and peaked after 90 min treatment (Figure S3D). The mRNA levels of *FRK1, WRKY22, CYP81F2,* and At2g17740 were much lower and less induced in the three *RTNLB4* O/E transgenic plants than in the wild-type after same T-pili treatment time (Figure S3A–D).

**Figure 6:**
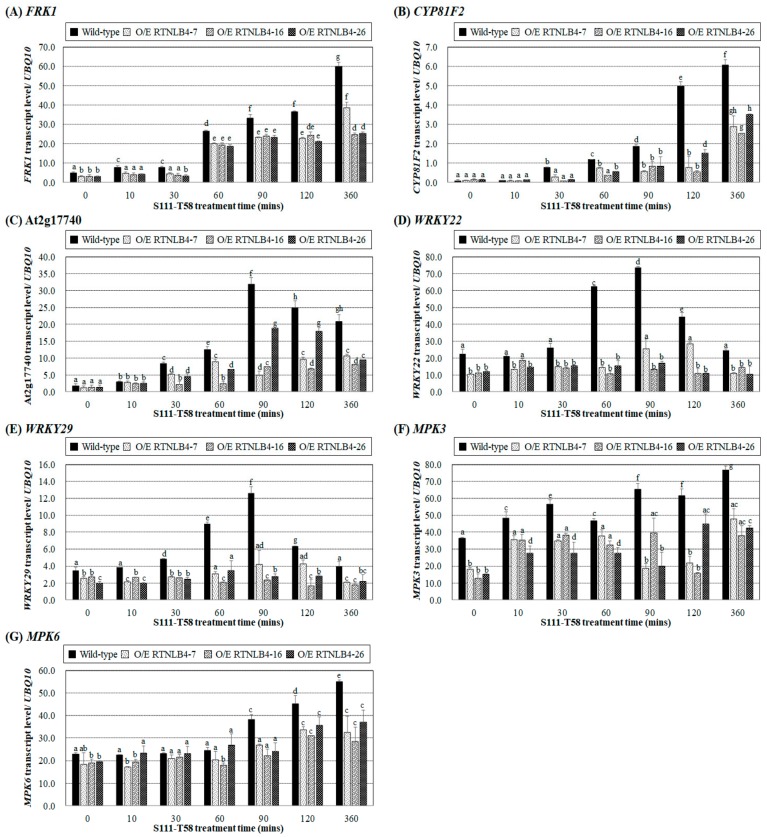
The VirB2 peptide, S111-T58, induced the expression of defense-related genes in wild-type plants but to a lesser extent in *RTNLB4* O/E transgenic plants. Gene expression of *FRK1* (**A**), *CYP81F2* (**B**), At2g17740 (**C**), *WRKY22* (**D**), *WRKY29* (**E**), *MPK3* (**F**), and *MPK6* (**G**) in seedlings of wild-type and *RTNLB4* O/E transgenic plants treated with 10 µM VirB2 peptide for 0, 10, 30, 60, 90, 120, and 360 min measured by qPCR analysis. The *UBQ10* (polyubiquitin 10) transcript level was an internal control. Data are mean ± SE from at least three independent biological experiments. Data were analyzed by Duncan tests and means with different letters were significantly different (*p* < 0.05).

We next treated wild-type and *RTNLB4* O/E transgenic plants with the VirB2 peptides S111-T58 or I63-I80 and analyzed the mRNA levels of the defense response genes *FRK1, CYP81F2,* and At2g17740. The mRNA levels of three genes gradually increased in wild-type seedlings and were relatively higher at later time points after treatment with the VirB2 peptides S111-T58 and I63-I80 (Figure 6A–C and Figure S4A–C). Genes encoding upstream transcriptional regulators WRKY22 and WRKY29 and signaling regulators MPK3 and MPK6 in the plant defense response signaling pathway were further examined after VirB2 peptide treatment. In the wild-type seedlings, S111-T58 treatment significantly increased *WRKY22* and *WRKY29* expression, which peaked after 90 min treatment (Figure 6D,E). In wild-type seedlings, I63–I80 peptide caused the highest level of *WRKY22* after 10 min treatment, which gradually decreased during 30 to 60 min treatments and was increased again after 90 min treatment (Figure S4D), whereas *WRKY29* level was gradually increased and peaked after 360 min treatment (Figure S4E). *MPK3* level was increased in wild-type seedlings after 30 min treatment with the two VirB2 peptides, decreased after 60 min treatment, and increased again after 90 min treatment (Figure 6F and Figure S4F). *MPK6* level was gradually increased in the wild type and peaked after 360 min treatment with the two VirB2 peptides (Figure 6G and Figure S4G). With VirB2 peptide treatment, the levels of seven selected genes and induction levels were lower in *RTNLB4* O/E transgenic seedlings than the wild type, which suggests that the mRNA levels of VirB2-induced plant defense genes were hampered (Figure 6A–G and Figure S4A–G).

On treating the wild type and three *rtnlb4* mutants with the two VirB2 peptides, the mRNA levels of *FRK1*, *CYP81F2*, At2g17740, *WRKY22*, and *WRKY29* were upregulated (Figures S5 and S6). In three *rtnlb4* mutants, the mRNA levels of the five selected genes were significantly diminished as compared with wild-type plants after treatment with the two VirB2 peptides (Figures S5 and S6). In addition, the *AtRbohD* gene, encoding a calcium-dependent NADPH oxidase, was induced after 10 min S111-T58 treatment and peaked after 360 min treatment (Figure S5D). In the three *rtnlb4* mutants, the levels of VirB2 peptide-elicited plant defense genes were diminished, which suggests that plant defense responses in *rtnlb4* mutants were less responsive to VirB2 peptides. Collectively, these data suggest that after VirB2 pretreatments, relatively lower mRNA levels of VirB2-induced plant defense gene expression may cause *RTNLB4* O/E transgenic plants and *rtnlb4* mutants to be more susceptible to *A. tumefaciens* infection.

To determine the possible signaling pathway elicited by VirB2 peptides, we treated *efr-1, fls2*, and *bak1* mutants with the VirB2 peptides to determine selected gene expression. *FRK1*, *CYP81F2*, At2g17740, *WRKY22*, and *WRKY29* were less upregulated in *efr-1, fls2*, and *bak1* mutants as compared with wild-type plants after treatment with the two VirB2 peptides (Figures S7 and S8). However, the levels of the seven tested genes were relatively higher in the *fls2* mutant than the other two mutants after treatment with the two VirB2 peptides (Figures S7 and S8). These data suggest that the VirB2 peptides may induce EFR- and BAK1-mediated plant defense responses, and the FLS2-elicited signaling pathway might play a minor role during VirB2 peptide elicitation.

### 2.7. Arabidopsis Seedling Growth Was Less Inhibited in RTNLB4 O/E Transgenic and rtnlb4 Mutant Plants after elf18 and VirB2 Peptide Treatments

**Figure 7:**
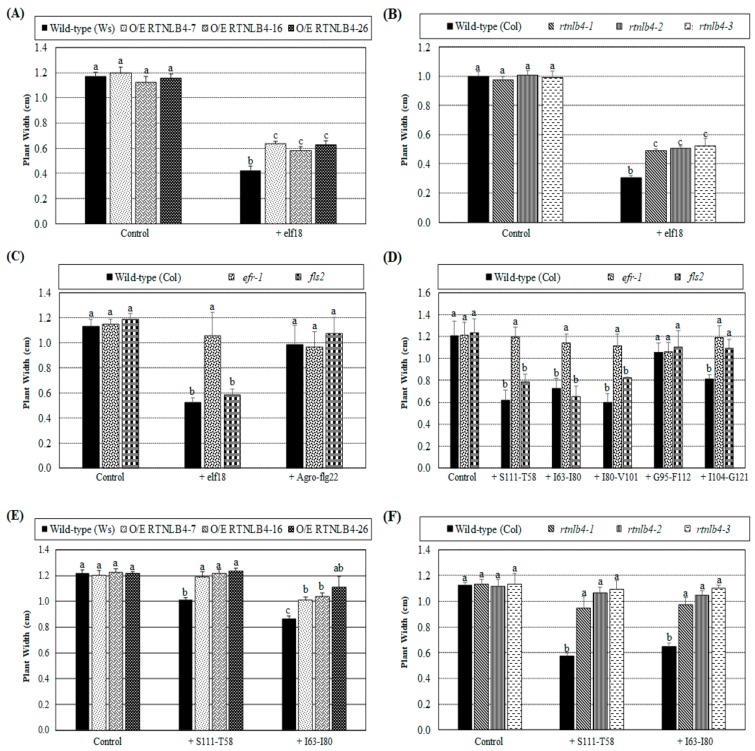
*Arabidopsis* seedling growth was inhibited by elf18 and VirB2 peptide treatments. Plant widths of wild-type, *RTNLB4* O/E transgenic (**A**), and *rtnlb4* mutant plants (**B**) were determined after treatment with 20 μM elf18 peptide or the mock control (dH_2_O) for 2 weeks. Plant widths of wild-type and *efr-1* and *fls2* mutants (**C**) were also measured after treatment with 20 μM elf18, Agro-flg22 or the mock control (dH_2_O). Five VirB2 peptides and the mock control (DMSO) were used to treat wild-type and *efr-1* and *fls2* mutants (**D**). Seedlings of wild-type, *RTNLB4* O/E transgenic (**E**) and *rtnlb4* mutant plants (**F**) were treated with two VirB2 peptides, S111-T58, or I63-I80, and the mock control (DMSO). Plant widths of these plants were determined after 2-week treatments. Data are mean ± SE from three independent experiments. More than 15 seedlings of each type plant for each treatment were examined in each independent experiment. Data were analyzed by Duncan tests and means with different letters were significantly different (*p* < 0.05).

Because elf18 and VirB2 peptides induced mRNA levels of plant defense genes, we next examined whether *Arabidopsis* seedling growth was affected by peptide treatments. The plant widths of wild-type plants (ecotypes: Columbia and Ws) with two weeks of elf18 peptide treatment were reduced to only 30% of wild-type widths with mock control treatment (Figure 7A,B). These data agreed with previous results showing that seedling growth was inhibited following elf18 treatments and that the genotypes of two ecotypes have no effect on seedling growth inhibition [33,58,59]. After elf18 peptide treatment, plant widths of *RTNLB4* O/E transgenic and *rtnlb4* mutant plants were reduced to 50% of the same type of plants with mock control treatment but were wider than wild-type plants with elf18 peptide treatment (Figure 7A,B). These data suggest that the less seedling growth inhibition in *RTNLB4* O/E transgenic and *rtnlb4* mutant plants as compared with wild-type plants might be due to diminished transcriptional activation of elf18-induced defense-related genes in transgenic and mutant plants. We also treated *efr-1* and *fls2* mutants with elf18 and Agro-flg22 peptides. As expected, the *efr-1* mutant showed comparable plant width with elf18 treatments as the same type of mutant under mock control, whereas the *fls2* mutant was sensitive to elf18 treatment and showed growth inhibition (Figure 7C). Agro-flg22 peptide was used as a negative control and caused no significant inhibition of seedling growth of wild-type and the two mutant plants (Figure 7C).

We next determined the seedling growth inhibition effects of five different VirB2 peptides in wild-type, *efr-1,* and *fls2* mutants. Plant widths of wild-type and the *fls2* mutant plants were shorter after treatment with the three VirB2 peptides S111-T58, I63-I80, and I80-V101 (Figure 7D). These data were consistent with data shown in Figure 5A, demonstrating that these VirB2 peptides had better ability than the VirB2 peptide G95-F112 to inhibit transient T-DNA expression in wild-type seedlings. The five VirB2 peptides caused no significant difference in plant width of *efr-1* mutants (Figure 7D), which suggests that the EFR protein may participate in the VirB2 peptide-inhibited seedling growth. Treatment with the two VirB2 peptides S111-T58 and I63-I80 caused no or a lesser degree of inhibition of seedling growth in *RTNLB4* O/E transgenic and *rtnlb4* mutant plants (Figure 7E,F), which indicates that the seedling growth inhibition caused by the VirB2 peptide was reduced in these plants.

### 2.8. H_2_O_2_ Accumulation Was Lower in RTNLB4 O/E Transgenic and rtnlb4 Mutant Plants after elf18 and VirB2 Peptide Treatment

**Figure 8:**
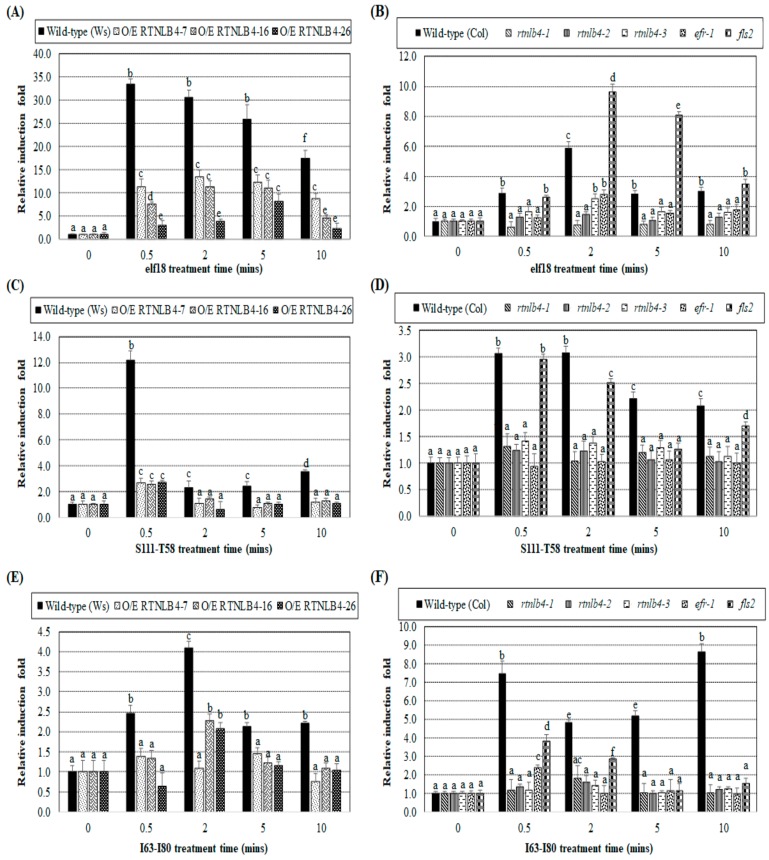
Induced H_2_O_2_ amounts were lower in *RTNLB4* O/E transgenic plants and *rtnlb4, efr-1,* and *fls2* mutants than in wild-type plants after with elf18 and VirB2 peptides. The H_2_O_2_ amount in wild-type, *RTNLB4* O/E transgenic plants (**A**) and three *rtnlb4*, *efr-1*, and *fls2* mutant plants (**B**) was determined at 0, 0.5, 2, 5, and 10 min after the addition of elf18. The H_2_O_2_ amount at each time was normalized to the H_2_O_2_ amount at 0 min. H_2_O_2_ amount in wild-type, *RTNLB4* O/E transgenic (**C**,**E**) and three *rtnlb4*, *efr-1*, and *fls2* mutant plants (**D**,**F**) was determined after adding two VirB2 peptides S111-T58 (C,D) or I63-I80 (E,F). Data are mean ± SE from more than 10 plants. Data were analyzed by Duncan tests and means with different letters were significantly different (*p* < 0.05).

To examine whether VirB2 peptide treatment could induce other plant defense responses, we determined hydrogen peroxide (H_2_O_2_) accumulation in leaves of wild-type, *RTNLB4* O/E transgenic and *rtnlb4, efr-1,* and *fls2* mutant plants after peptide treatments. The H_2_O_2_ amounts in various plants were measured at 0, 0.5, 2, 5, and 10 min after the addition of elf18 or VirB2 peptides S111-T58 and I63-I80. The H_2_O_2_ amount at each time was normalized to the H_2_O_2_ amount at 0 min. H_2_O_2_ accumulation in both Ws and Columbia wild-type plants was significantly increased at 0.5 min after elf18 treatment and decreased at a later time point, 10 min post-treatment (Figure 8A,B), which is consistent with previously published data [33]. H_2_O_2_ level in wild-type, both Ws and Columbia, did not differ significantly after treatment with dH_2_O for elf18 and DMSO for the VirB2 peptides, respectively (Figure S9). After elf18 treatment, H_2_O_2_ level in *RTNLB4* O/E transgenic and *rtnlb4* and *efr-1* mutant plants was less induced as compared with the corresponding wild-type plants at the same treatment time (Figure 8A,B). Only in the *fls2* mutant was H_2_O_2_ at a comparable level or higher than the wild-type level at the same post-elf18 treatment time (Figure 8B).

Treatment with the two VirB2 peptides S111-T58 and I63-I80 caused H_2_O_2_ induction at an early time, 0.5 min after treatment, in the two wild-type ecotypes, and induction levels became lower at later times (Figure 8C–F). The slightly different H_2_O_2_ induction profiles in Ws and Columbia wild-type plants after elf18 and VirB2 peptide treatments might be due to the difference in the two ecotypes (Figure 8A–F). Similar to results obtained with elf18 treatments (Figure 8A,B), H_2_O_2_ induction was lower in RTNLB4 O/E transgenic and rtnlb4 and efr-1 mutant plants than in corresponding wild-type plants at the same time after two VirB2 peptide treatments (Figure 8C–F). The H_2_O_2_ induction was higher in the fls2 mutant than the efr-1 mutant after treatment with the two VirB2 peptides (Figure 8D,F). Collectively, these data show that elf18- and VirB2 peptide-induced H_2_O_2_ production was impeded when the RTNLB4 level was overexpressed in transgenic plants or reduced in mutants. Additionally, the VirB2-induced H_2_O_2_ production mainly required proper functioning of the EFR protein.

## 3. Discussion

Plants have developed a complicated immune system that is activated upon detecting various PAMPs or MAMPs by membrane-associated PRRs to hinder pathogen infections. The PAMP-triggered immune response includes activation of MAPK signaling cascades, transcriptional reprogramming mediated by WRKY transcription factors, ROS burst, and other defense responses. The *Agrobacterium*-derived PAMP, elf18 peptide, is recognized by the *Arabidopsis* receptor kinase protein EFR protein. In this study, we observed that when *RTNLB4* level was abnormal in plants, the elf18-induced plant defense responses were alleviated, so RTNLB4 may be involved in the elf18-derived immune response. During *A. tumefaciens* infection, Vir proteins and T-DNA are transferred via a T4SS consisting of a transmembrane transporter and a filamentous pilus (T-pilus). The T-pilus mainly consists of cyclized VirB2 proteins. Here, we showed that VirB2 peptide treatment stimulated the expression of defense-related genes and H_2_O_2_ production and caused seedling growth inhibition. The VirB2 peptide-derived defense-related response was dampened in the *efr-1, rtnlb4* mutants, and *RTNLB4* O/E transgenic plants. Thus, RTNLB4 protein may participate in *A. tumefaciens* VirB2 peptide-induced plant immunity, and the VirB-encoded pilus may have additional roles other than T-DNA and Vir protein transfer.

### 3.1. RTNLB4 Plays a Role in Plant Defense Responses and Affects A. tumefaciens Infection

The reticulon (RTN) protein family contains a highly conserved C-terminal region called reticulon homology domain (RHD), which consists of a hydrophilic region sandwiched by two hydrophobic regions. The RHD domain forms a transmembrane structure on the ER, to help with ER tubular structure formation [39]. Previous studies have demonstrated that RTNLB1 and -2 proteins are involved in FLS2-activated signaling and immunity [43]. The reduction of both *RTNLB1* and *-2* or excess *RTNLB1* may perturb the efficient FLS2 transport to the PM and consequently weaken FLS2 activity [43]. RTNLB1, -2, -3, -4, and -8 interact with themselves, each other, and with *A. tumefaciens* VirB2; and have major roles during *A. tumefaciens* infection [40,41,42,44]. The soybean RHD protein (GmRHP) affects viral infection by interacting with *Soybean mosaic virus*-encoded P3, which is an essential factor for viral replication complex [60]. These previous studies revealed possible involvement of RHD domain-containing proteins in plant–pathogen interactions.

Here, we have uncovered a possible link between *RTNLB4* gene expression and the *A. tumefaciens* elf18 peptide-derived plant defense response. *RTNLB4* expression was induced in wild-type plants after treatment with *A. tumefaciens* elf18 peptide. Furthermore, levels of *A. tumefaciens* elf18-induced defense response genes were reduced in both *RTNLB4* O/E transgenic and *rtnlb4* mutant plants, which suggests that an abnormal level of *RTNLB4* in plants may impair elf18-mediated plant immunity. Thus, *RTNLB4* might participate in the *A. tumefaciens* elf18-induced plant immune response. This hypothesis was further supported by results showing that after elf18 peptide pretreatment, *A. tumefaciens*-mediated transient transformation efficiency was more decreased in wild-type plants than in *RTNLB4* O/E transgenic and *rtnlb4* mutant plants. Of note, without *A. tumefaciens* elf18 peptide elicitation, the mRNA levels of defense-related genes were significantly lower in only *RTNLB4* O/E transgenic but not in *rtnlb4* mutant plants than wild-type plants. These data may suggest that elf18 peptide-induced defense gene expression levels were affected more than basal defense gene expression levels by the abnormally high or low level of *RTNLB4* in plants. These observations were consistent with previous studies showing reduced flg22-induced resistance of *Pst* DC3000 in the *rtnlb1rtnlb2* mutant and *RTNLB1* O/E transgenic plants [43]. Our studies may also indicate that the RTNLB protein family might participate in the EFR-mediated downstream signaling pathways.

The RTN proteins reside mainly in the ER and plasma membrane (PM) [39,61] and participate in intracellular protein trafficking, vesicle formation, and membrane curvature [37,38]. Plant cells utilize secretory and endocytic membrane trafficking systems to deliver surface-localized immune receptors, antimicrobial compounds, and defense proteins [35,36,62]. Inside cells, proteins are first synthesized in the ER, exported to the Golgi apparatus and then the trans-Golgi network (TGN) by vesicle trafficking, and finally trasnsported to various subcellular membrane compartments or the extracellular space. The PM-located PRRs, such as FLS2 and EFR, are maintained at certain levels at the PM for pathogen detection by endocytic trafficking and recycling [62]. In view of the RTNLB roles in intracellular protein trafficking, it is highly possible that the RTNLB4 protein, similar to known functions of RTNLB1 and -2 [43], play major roles in export and/or endocytic recycling of PM-located PRRs, including FLS2 and EFR. Either reduction or excess of the RTNLB4 protein in plant cells may perturb the stoichiometry of components involved in transport pathway of PM-located PRRs, and subsequently affected PRR-mediated downstream signaling pathways. Furthermore, two PRRs, FLS2 and the fungal PAMP chitin receptor LYSIN MOTIF DOMAIN-CONTAINING GLYCOSYLPHOSPHATIDYLINOSITOL-ANCHORED PROTEIN 2 (LYM2), can localize at specific locations of PM, the plasmodesmata, the formation of which is regulated by RTNLB proteins [45,63]. These data further support the positive link between RTNLB functions and PRR-induced plant immune responses.

Upon pathogen infection, several ER stress-related genes are upregulated to help boost plant immunity, which includes regulation of ER protein-folding quality control, antimicrobial protein secretion, and induction of programmed cell death [64]. In mammals, RTN1A is involved in mediating ER stress in kidney tubular cells, which suggests a correlation between RTN protein levels and ER stress [65]. Moreover, the RTN protein in yeast, YOP1, regulates the ER inheritance block during ER stress [66]. These observations in mammals and yeast suggest a possible connection between ER-resided RTNLB proteins and ER stress signaling pathways in plant immunity.

Unexpectedly, we observed that defense-related gene expression levels were not significantly different between rtnlb4 mutant and wild-type plants in the absence of elf18 peptide treatments. The rtnlb4 mutants were more recalcitrant to A. tumefaciens infection than wild-type plants. Therefore, RTNLB4 may affect other aspects of A. tumefaciens transformation in addition to its effect on plant defense-related gene expression levels. During A. tumefaciens infection, cytoplasmic trafficking and nuclear targeting of VirE2 protein require the host endocytosis system and are facilitated by host ER/actin networks [8,9,10]. The RTNLB proteins mainly localize in the ER and PM and participate in intracellular protein trafficking and vesicle formation [37,38,39,61]. Therefore, it is possible that RTNLB proteins might affect A. tumefaciens transformation process by affecting intracellular vesicle formation or the transferring process.

### 3.2. Elf18 and VirB2 Peptides May Induce a Common Set of Plant Defense Responses

The export apparatus of T-DNA and virulence proteins in *A. tumefaciens* consists of two major structural components: T-pilus and a membrane-associated transporter encoded by *virB* and *virD4* genes of the Ti plasmid [4,5]. T-pilus mainly consists of the processed and cyclized VirB2 protein [48]. Because the VirB2 interacting protein, RTNLB4, may be involved in *A. tumefaciens* elf18-induced plant immune responses, we examined in this study whether the VirB2 peptide might be similar to other known bacteria PAMPs that can induce plant immunity.

Products of the *virB* operon are required for tumorigenesis [67]. Genetic studies revealed the importance of VirB2 for *A. tumefaciens* virulence [49,50]. However, mutational analysis isolated several uncoupling mutants in the VirB2, VirB6, VirB9, VirB10, and VirB11 that prevent T-pilus assembly but not substrate translocation [52,68,69,70]. The filamentous T-pilus might play less important roles in tumorigenesis but may have more critical roles in the transient transformation of *Arabidopsis* seedlings [52]. Thus, in this study, we used *A. tumefaciens*-mediated transient transformation assays with *Arabidopsis* seedlings to examine whether pretreatment with VirB2 peptides could affect subsequent transformation efficiency. VirB2 is translated as a 12.3-kD pro-pilin protein but is processed to a 7.2-kD pilin protein which may associate with the bacterial inner membrane via two transmembrane domains. T-pilin, which is 74 amino acid residues long, is coupled between the amino terminal Gln-48 residue to the Gly-121 residue at the carboxy terminus in a head-to-tail peptide bond, thus forming the unusual cyclic peptide [48].

Here, we have designed five VirB2 peptides consisting of two transmembrane domains, a cytoplasmic domain between two transmembrane domains, a C-terminal periplasmic domain, and a C-terminus domain connected with the N-terminus (Table 1). Pretreating *Arabidopsis* with three VirB2 peptides, S111-T58, I63-I80, and I80-V101, conferred the highest reduction in transient transformation rates. Consistently, two VirB2 peptides, S111-T58 and I63-I80, showed the typical characteristic of known PAMP-induced immunity, which includes defense-related gene expression, H_2_O_2_ production, and *Arabidopsis* seedling growth inhibition. Thus, these VirB2 regions, which include amino acid sequences from Gln-48 residue to Val-101 residue, might be important for plant–*A. tumefaciens* interactions. These results agree with those obtained from mutant analysis of VirB2 revealing that Pro-56, Gly-69, Phe-71, Ile-80, Met-88, Phe-89, and Arg-91 residues are crucial for extracellular VirB2 production and *A. tumefaciens* virulence [52]. Following the perception of various PAMPs, including flg22, elf18, chitin, and peptidoglycan, the MPK3/6-mediated signaling cascade is activated, which leads to induction of genes encoding WRKY transcription factors (WRKY22 and WRKY29) and other defense-related proteins [22,27,32,33]. In addition to post-translational modification-mediated regulation of MAPK activities, several studies have shown that MPK3 transcript levels are increased under peptide and chemical treatments or stress [71,72,73,74]. Consistently, in this study, we observed increased expression of several early defense genes, including *MPK3, MPK6, WRKY22, WRKY29, FRK1*, and *PR1*. Most of these genes were induced as early as 10 min after peptide treatments, and some gradually increased their expression during treatment, which is consistent with previous results showing that several genes were upregulated in *Arabidopsis* at 1 hr after elf18 and flg22 peptide elictation [33,74]. One notable result is that *efr* mutants treated with *A. tumefaciens* crude extracts still showed defense responses, which implies that other unidentified PAMPs of *A. tumefaciens* can be detected by plants [33]. In agreement with this observation, we detected the induction of several defense-related genes, including *MPK3, MPK6, WRKY22, WRKY29, FRK1, CYP81F2*, and At2g17740, after treatment with the two VirB2 peptides. Similarly, *A. tumefaciens* T-pili induced *WRKY22, FRK1, CYP81F2*, and At2g17740 expression. These observations suggest that *A. tumefaciens* T-pilus and/or VirB2 peptides may be detected by plant cells and activate downstream defense responses. *CYP81F2*, encoding a cytochrome P450 monooxygenase, is essential for the pathogen-induced accumulation of 4-methoxyindol-3-ylmethylglucosinolate, which is activated by the PEN2 myrosinase for antifungal defense and is also induced by *A. tumefaciens* infection [75,76]. Furthermore, several genes involved in indole glucosinolate (iGS) modification were upregulated during *A. tumefaciens* infections, indicating the direct involvement of glucosinolate in *A. tumefaciens* infection [76]. The importance of the At2g17740 gene product in *A. tumefaciens* infection awaits further investigation.

PAMP-triggered immunity (PTI) includes ROS burst, MAPK activation, and transcriptional reprogramming, which inhibits seedling growth [22]. In this study, VirB2 treatment induced H_2_O_2_ within 30 sec, but the accumulation was relatively lower after VirB2 peptide treatment as compared with elf18 peptide treatment, which suggests that the elf18 peptide may induce stronger defense responses than VirB2 peptides. In plants, defense activation usually comes at the expense of plant growth. Thus, we also observed that the two VirB2 peptides inhibited *Arabidopsis* seedling growth. All the VirB2 peptide-induced defense responses were diminished to a greater degree in the *efr-1* than *fls2* mutant, which suggests that the EFR-mediated defense response may overlap with VirB2 peptide-derived defense responses. In *RTNLB4* O/E transgenic and *rtnlb4* mutant plants, the VirB2 peptide-induced defense-related gene expression, H_2_O_2_ accumulation, and seedling growth inhibition were all affected, so a proper level and/or function of RTNLB4 in plant cells may contribute to the plant defense responses induced by VirB2 peptide.

From our study, it is possible that *A. tumefaciens* T-pilus and/or VirB2 peptides may have additional roles besides substrate transfer. During *A. tumefaciens* infection, plant cells may detect bacteria-derived PAMPs such as elf18 and VirB2 peptides and activate the plant immune response with the help of RTNLB4 and EFR-mediated MAPK signaling.

## 4. Materials and Methods 

### 4.1. Generation of RTNLB4 and T7-tagged-RTNLB4 Overexpression (O/E) Arabidopsis Thaliana Transgenic Plants

To generate *Arabidopsis RTNLB4* and T7-tagged-RTNLB4 O/E transgenic plants, we used a binary vector, pE1798. pE1798 contained a double Cauliflower mosaic virus (CaMV) 35S promoter, a *Nos* (nopaline synthase) terminator, and a hygromycin resistance (*hptII*) gene as a selection marker in the T-DNA region [42,44]. The PCR products containing the coding sequences of the *RTNLB4* were obtained with *Arabidopsis* cDNA used as templates, high-fidelity Phusion DNA polymerase, and appropriate primers (Table S1). The PCR products were digested with *Spe*I and *BamH*I, then cloned into the pBluescript plasmid to create pBluescript-RTNLB4 (Table S2) and confirmed by sequencing. The *Xba*I-*Kpn*I fragment from the pBluescript-RTNLB4 was then cloned into the same sites of the pE1798 plasmid (Table S2).

To overexpress the T7-tagged-RTNLB4 in *Arabidopsis* transgenic plants, the *Xba*I-*Kpn*I fragment from the plasmid pET23a-RTNLB4 [42] was then cloned into the same sites of the pE1798 plasmid to create the plasmid pE1798-T7-tag-RTNLB4 (Table S2). These pE1978 series plasmids were separately transformed into the disarmed strain *A. tumefaciens* GV3101(pMP90) [77] to generate *Arabidopsis* O/E transgenic plants by a floral dip method [78].

### 4.2. DNA Isolation from Arabidopsis Plants and Genomic DNA PCR Analysis

The *Arabidopsis* T-DNA insertion mutants *rtnlb4-1, rtnlb4-2, and rtnlb4-3* (ecotype: Columbia CS60,000) were identified by using the SIGnAL T-DNA Express *Arabidopsis* Gene Mapping Tool (http://signal.salk.edu/) [79]. The *rtnlb4* mutant seeds were acquired from the *Arabidopsis* Biological Resource Center (ABRC; Ohio State University, Columbus, OH, USA). Leaves of 3-week-old seedlings of *rtnlb4* mutants grown in Gamborg’s B5 medium (PhytoTechnology Laboratories, Carlsbad, CA, USA) were used to isolate genomic DNA as described [80]. A PCR-based approach and the SIGnAL T-DNA Express Gene Mapping Tool (http://signal.salk.edu/) were used to determine the homozygosity of *Arabidopsis rtnlb4* according to Alson et al. 2003 [79]. Primers for genomic DNA PCR analysis are in Table S1. The PCR reaction was performed in a 50 μL reaction volume with 2 units of GenTaq polymerase (GMbiolab Co., Taichung, Taiwan), a 2.5 mM dNTP mixture, 1× Taq polymerase reaction buffer, and 0.25 μm PCR primers. The PCR amplification cycle was 95 °C for 1 min (1 cycle); 94 °C for 30 s, 56 °C for 40 s, 72 °C for 1 min (30 cycles) and 72 °C for 5 min (1 cycle).

### 4.3. RNA Isolation from Arabidopsis Plants and Quantitative Real-Time PCR (qPCR) Analysis

The 10-day-old seedlings of wild-type plants (ecotypes: Columbia and Wassilewskija [Ws]), *efr-1* [33], *fls2* [25], *bak1* [81], and *rtnlb4* mutants (ecotype: Columbia CS60,000), and *RTNLB4* O/E transgenic plants (ecotype: Ws) grown on Gamborg’s B5 medium using 16-h-light/8-h-dark conditions at 24 °C were pressure infiltrated [32] with 10 μM of various peptides (elf18 or VirB2 peptides; Table 1) or T-pili for different times (0, 10, 30, 60, 90, 120, or 360 min) and collected for RNA isolation. Additionally, RNA was isolated from tissues from 4- to 5-week-old uninfected wild-type plants (ecotypes: Columbia and Ws), *rtnlb4* mutants, and *RTNLB4* O/E transgenic plants. Plant tissues were ground with liquid nitrogen and mixed with TRIZOL LS reagents (Invitrogen, Carlsbad, CA, USA) according to the manufacturer’s instructions. Then, 1-3 μg RNA was treated with DNase I (Thermo Fisher Scientific, Waltham, MA, USA) to remove any DNA contamination according to manufacturer’s instructions.

cDNA was obtained by reverse transcription of 1 μg RNA samples by using oligo-dT primers. The 100 ng cDNAs were used for quantitative real-time PCR (qPCR) with the IQ^2^ SYBR Green Fast qPCR System Master Mix (Bio-genesis Technologies Inc., Taipei, Taiwan) in a MS3000P QPCR system (Agilent Technologies, Santa Clara, CA, USA). Primers used for qPCR are in Table S1. The *UBQ10* (polyubiquitin 10) transcript level was an internal control for each qPCR reaction. The PCR amplification cycle was 99 °C for 1 min (1 cycle); 94 °C for 30 s, 56 °C for 40 s, 72 °C for 1 min (50 cycles); 99 °C for 1 min (1 cycle); 55 °C for 3 min (1 cycle); and 95 °C for 30 s (1 cycle). More than 3 independent real-time PCR reactions were performed with RNA samples isolated from at least 8-10 different *Arabidopsis* plants.

### 4.4. Protein Extraction from Arabidopsis Plants and Protein Gel Blot Analysis

Protein crude extracts of *RTNLB4* O/E transgenic seedlings were isolated by using CelLytic P (Sigma, St. Louis, MO, USA) and a protease inhibitor cocktail (1:100 dilution) from Sigma according to the manufacturer’s instructions. Protein extract concentrations were determined with a BCA protein assay kit (Pierce, Rockford, IL, USA) and a spectroscopy (PARADIGM Detection Platform, Beckman Coulter Inc., Indianapolis, IN, USA). Equal amounts of plant proteins were analyzed in 12.5% SDS-polyacrylamide gels. Protein gel blot analyses were then performed with a 1:1000 dilution of T7-tag antibody (Abcam, Cambridge, UK), then with a 1:20,000 dilution of horseradish peroxidase-conjugated goat anti-rabbit IgG antibody (PerkinElmer Life and Analytical Sciences, Boston, MA, USA). The membranes were developed by chemiluminescent detection (PerkinElmer Life and Analytical Sciences, Boston, MA, USA) and X-ray-films were used to capture signals.

### 4.5. Agrobacterium Tumefaciens-Mediated Stable, Transient Root and Seedling Transformation Assays of rtnlb4 Mutant Plants and RTNLB4 O/E Arabidopsis Transgenic Plants

For *A. tumefaciens*-mediated root transformation assays, seeds of *rtnlb4* mutants, and *RTNLB4* O/E T4 generation transgenic plants were surface-sterilized and grown on Gamborg’s B5 medium using 16-h-light/8-h-dark conditions at 24 °C with the appropriate antibiotics (kanamycin 50 μg mL^−1^ for mutants and hygromycin 20 μg mL^−1^ for overexpression transgenic plants) for 10–14 days. Seedlings were then grown on B5 medium in baby food jars without antibiotics for 3-4 weeks to perform stable and transient root transformation assays according to previous studies [42,44]. *A. tumefaciens* strains (Table S2) were cultured in 523 medium [82] with appropriate antibiotics (rifampicin 50 μg mL^−1^, kanamycin 20 μg mL^−1^) at 28 °C. Overnight-grown bacterial cultures were subcultured into 523 medium with antibiotics and grown to 10^9^ colony forming unit (cfu) mL^−1^. Bacterial cells were washed with and resuspended in 0.9% sodium chloride at 10^5^, 10^6^, or 10^8^ cfu mL^−1^ for root transformation assays.

For stable root transformation assays, root segments were transferred onto MS medium and co-incubated with a tumorigenic strain of *A. tumefaciens*, A208 (Table S2), at 22 to 24 °C. After 2 days of infection, root segments were separated and transferred to MS medium (without plant hormones) containing antibiotic timentin (100 μg mL^−1^) for 1 month to score tumor formation rates. For transient root transformation assays, root segments were co-incubated with an *A. tumefaciens* At849 strain containing the pBISN1 binary vector (Table S2) for 2 days. After infection, root segments were transferred on the callus induction medium (CIM) with timentin at 22 to 24 °C [42,44]. After 4 days, roots were stained with X-gluc (5-bromo-4-chloro-3-indolyl beta-D-glucuronic acid) staining solutions at 37 °C for 1 day. Roots were observed with a stereoscopic microscope to obtain transient transformation rates. For root transformation assays, 15–20 different *Arabidopsis* plants were infected with each *A. tumefaciens* strain and 60–80 root segments were examined for each plant for each independent transformation assay.

The transient seedling transformation assays (AGROBEST) were performed as described [44,47]. *Arabidopsis* seeds were germinated in a 6-well plate with the 1/2 MS medium (pH5.7) and 0.5% sucrose at 22 to 24 °C for 7 days. The *A. tumefaciens* C58C1(pTiB6S3ΔT) strain with a pBISN1 binary vector (Table S2) was grown in 523 medium with the appropriate antibiotics (rifampicin 50 μg mL^−1^, kanamycin 20 μg mL^−1^) at 28 °C for 16 hr. Bacterial cultures were further grown at 28 °C in acidic AB-MES medium with 200 μM acetosyringone (AS) for 24 hr. After AS treatment, bacterial cells were washed and resuspended in infection solutions at 10^7^ cfu mL^−1^ for seedling transformation assays.

Then, 10 μM of various peptides (Table 1) were used to pressure infiltrated seedlings 6 hr before bacterial infection [32]. The elf18 and Agro-flg22 were dissolved in dH_2_O, and dH_2_O was used as the mock control in seedling transformation assays. Tested VirB2 peptides were dissolved in DMSO solution, and DMSO was used as the mock control in assays. *Arabidopsis* seedlings were then infected with AS-induced bacteria cells at 22 to 24 °C for 3 days. After infection, seedlings were ground with liquid nitrogen and mixed with extraction buffers for fluorescent 4-methylumbelliferyl-β-D-glucuronide (MUG) assays as described [47]. The fluorescence was determined by using a 96 microplate reader (PARADIGM Detection Platform) at 365 nm excitation and 455 nm emission. The BCA protein assay kit and spectroscopy were used to determine protein concentration for each protein sample. The relative GUS activity was the fluorescence signal normalized by an equal amount of proteins. About 15–20 *Arabidopsis* seedlings were infected with *A. tumefaciens* for each independent transformation assay, and more than 3 independent transformation assays were conducted. The transformation rates were average values from at least three independent experiments. Error bars were calculated by using the Excel STDEVP function. The significance test between treatments was based on a pairwise Student t-test or a Duncan test (*p* < 0.05).

### 4.6. Isolation of Pili of A. tumefaciens

Pili of the *A. tumefaciens* strain C58 (Table S2) were isolated according to Lai and Kado, 1998 [83] with minor modifications. Bacteria were grown in acidic AB-MES medium to mid-log phase, and bacteria culture was then spread on solidified AB-MES (pH 5.5) medium (with 200 μM AS) and incubated at 19 °C for 3 days. Bacteria cells were scraped off by using an L-glass rod, resuspended in 10 mM sodium phosphate buffer (pH 5.3), and collected by centrifugation. The bacterial appendages were released by passing bacteria solutions through a hypodermic needle (26 gauge) for five times on ice. Bacteria cells were removed by centrifugation, and supernatant was filtrated by using a syringe filter with 0.2 µm pore size (Sartorius, Goettingen, Germany). Pili were collated by centrifugation at 100,000×*g* for 3 h at 4 °C and resuspended in buffer B (10 mM Tris-HCl (pH 7.5), 100 mM NaCl, and 0.5% sodium deoxycholate). Concentrations of pili extract were determined by absorbance measured at 280 nm (A_280_) by comparison with a standard curve of the bovine serum albumin (BSA). Pili extract was fragmented by sonication and used to treat *Arabidopsis* seedlings for different times (0, 10, 30, 60, 90, 120, or 360 min).

### 4.7. Seedling Growth Inhibition Assays

*Arabidopsis* seeds of wild-type, *efr1*, *fls2*, *rtnlb4* mutants, and *RTNLB4* O/E transgenic plants were surface-sterilized and grown in 6-well plate at 22 to 24 °C (16 light/ 8 dark) with 1/2 MS liquid medium (pH 5.7) and 0.5% sucrose. Then 20 μM of various peptides (Table 1) was used to treat seedlings [33]; seedlings were photographed, and the plant width was measured by using Image J after 2 weeks of treatment.

### 4.8. Hydrogen Peroxide (H_2_O_2_) Detection by Ferrous Oxidation Xylenol Orange (XO) Assays

Leaves from 4- to 5-week-old wild-type plants; *efr-1*, *fls2*, *bak1*, and *rtnlb4* mutants; and *RTNLB4* O/E transgenic plants were used for infiltration with 10 μM of various peptides (Table 1). Leaf tissue was collected to determine hydrogen peroxide (H_2_O_2_) amounts at 0, 0.5, 2, 5, and 10 min post-treatment. H_2_O_2_ amounts were determined according to Jiang et al. (1990) [84] with minor modifications. Leaf tissue was first ground with 50 mM phosphate buffers (pH 7.0). After centrifugation, 900 µL of the working solution (250 µM Fe_2_SO_4_·7H2O, 25 mM H_2_SO_4_, and 100 µM xylenol orange) was added to 100 µL of the supernatant. H_2_O_2_ was quantified by colorimetric reaction with xylenol orange (XO). Concentrations of H_2_O_2_ were determined by absorbance measured at 560 nm (A_560_) by comparison with a standard curve of 0-100 µM H_2_O_2_ [85,86,87,88,89,90,91].

## Figures and Tables

**Table 1:** Related information of peptides used in this study.

Peptide Names	Peptide Position of the VirB2 Protein	Peptide Sequences	Length (a. a.)	pI	Hydrophobicity	Mw (Da)	References
VirB2-S111-T58	C-terminus connected with N-terminus	S^111th^FLGKTLTGGGQSAGGGTDPAT^58th^	22	5.55	27.3 %	1980.12	This study
VirB2-I63-I80	Transmembrane domain (TM) 1	I^63th^CTFILGPFGQSLAVLGI^80th^	18	5.52	61.1 %	1849.26	This study
VirB2-I80-V101	Part of TM1, region between 2 TM domains, part of TM2	I^80th^VAIGISWMFGRASLGLVAGVV^101th^	22	9.75	68.2 %	2216.71	This study
VirB2-G95-F112	Transmembrane domain (TM) 2	G^95th^LVAGVVGGIVIMFGASF^112th^	18	5.52	66.7 %	1694.06	This study
VirB2-I104-G121	C-terminal region	I^104th^VIMFGASFLGKTLTGGG^121th^	18	8.75	50.0 %	1769.13	This study
Elf18	N-terminal region	M^1st^SKEKFERTKPHVNVGTI^18th^	18	9.70	33.3 %	2101.45	[51]
Agro-Flg22	N-terminal region	S^19th^RVSSGLRVKSASDNAAYWSIA^40th^	22	9.98	36.4 %	2325.57	[24]

Red letters represent hydrophobic uncharged residues. The order of amino acid in the VirB2 peptide was based on Wu et al. 2014 [52]. pI value and molecule weight (Mw) of each peptide were obtained using the Compute pI/Mw program [53]. Hydrophobicity value of each peptide was calculated using the PEPTIDE 2.0 program [54].

## Supplementary Materials

Supplementary materials can be found at https://www.mdpi.com/1422-0067/21/5/1722/s1.
