# Supplementary material for: Arabidopsis RETICULON-LIKE4 (RTNLB4) Protein Participates in Agrobacterium Infection and VirB2 Peptide-Induced Plant Defense Response
Source: Int J Mol Sci. 2020 Mar 3;21(5):1722. doi: 10.3390/ijms21051722 (PMC7084338; doi:10.3390/ijms21051722)
Supplement: Supplementary file 1 [file ijms-21-01722-s001.zip › Suppl figure and table/Figure S1-T7-4OE-final.docx]

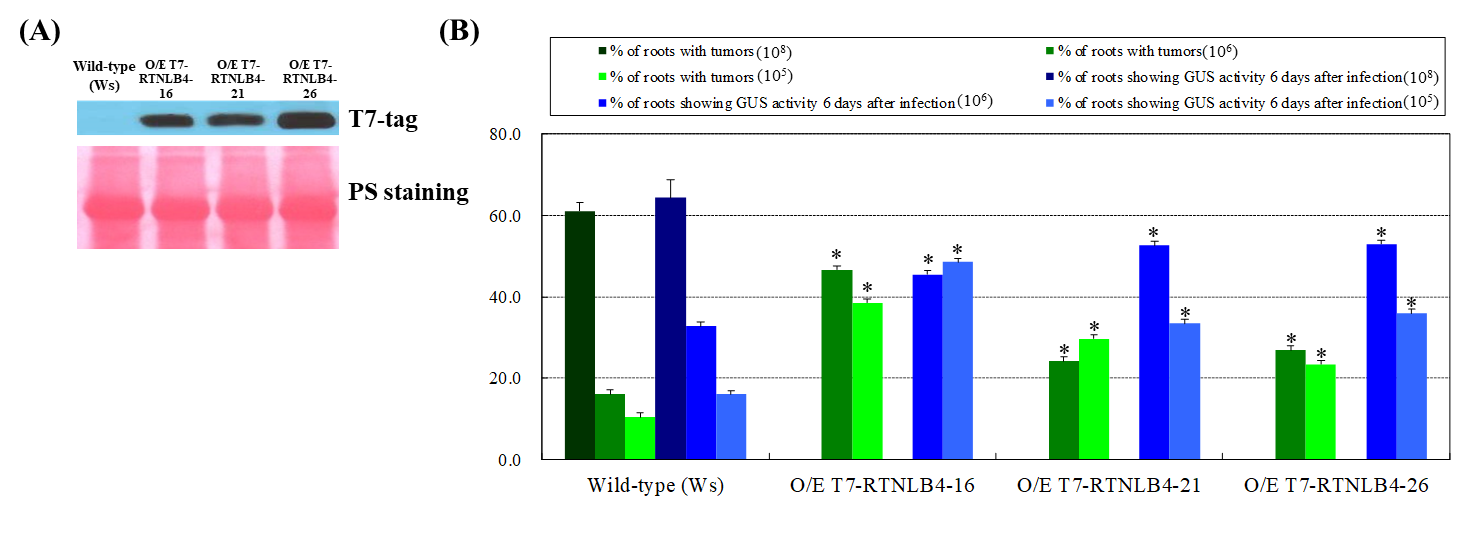


**Figure S1**. T7-tagged-*RTNLB4* overexpression (O/E) transgenic plants were hypersusceptible to *A. tumefaciens* infections. A) Western blot analysis of protein samples as shown in (A) using an antibody against T7 tag. Ponceau S (PS) staining was used to show equivalent loading of total protein in each lane. B) Stable and transient transformation efficiency of T7-tagged-*RTNLB4* O/E and wild-type plants. Green bars show the percentage of root segments with tumors after infection with 10^8^, 10^6^, or 10^5^ cfu mL^-1^ *A. tumefaciens* A208. Blue bars indicate the percentage of root segments with GUS activity after infection with 10^8^, 10^6^, or 10^5^ cfu mL^-1^ *A. tumefaciens* At849 strain. Data are mean±SE transformation results from more than 15 plants. At least 80 root segments were examined for each plant. ＊ P<0.05 compared with the wild-type by pairwise Student’s *t* test.
