# Supplementary material for: Arabidopsis RETICULON-LIKE4 (RTNLB4) Protein Participates in Agrobacterium Infection and VirB2 Peptide-Induced Plant Defense Response
Source: Int J Mol Sci. 2020 Mar 3;21(5):1722. doi: 10.3390/ijms21051722 (PMC7084338; doi:10.3390/ijms21051722)
Supplement: Supplementary file 1 [file ijms-21-01722-s001.zip › Suppl figure and table/Figure S2-4 mutant elf-final.docx]

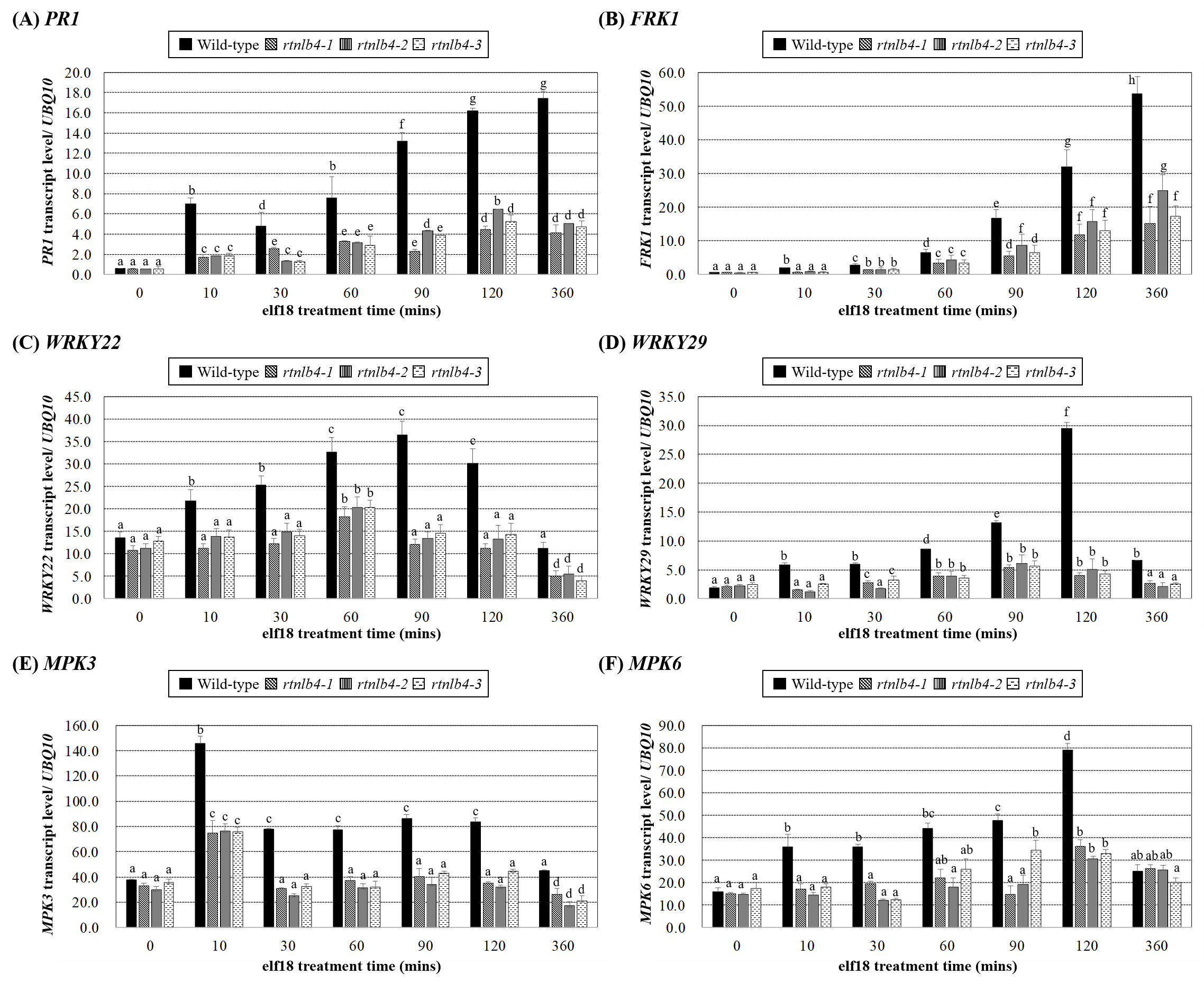


**Figure S2**. Expression of defense genes was less induced by elf18 in *rtnlb4* mutants. mRNA levels of *PR1* (A), *FRK1* (B), *WRKY22* (C), *WRKY29* (D), *MPK3* (E), and *MPK6* (F) in seedlings of *rtnlb4* mutants and wild-type plants treated with 10 µM elf18 for 0, 10, 30, 60, 90, 120, and 360 min measured by qPCR analysis. The *UBQ10* (polyubiquitin 10) transcript level was an internal control. Data are mean±SE from at least 3 independent biological experiments. Duncan tests were used for statistical analysis and means with different letters were significantly different (P < 0.05).
