# Supplementary material for: Arabidopsis RETICULON-LIKE4 (RTNLB4) Protein Participates in Agrobacterium Infection and VirB2 Peptide-Induced Plant Defense Response
Source: Int J Mol Sci. 2020 Mar 3;21(5):1722. doi: 10.3390/ijms21051722 (PMC7084338; doi:10.3390/ijms21051722)
Supplement: Supplementary file 1 [file ijms-21-01722-s001.zip › Suppl figure and table/Figure S4-4OE I63I80-f.docx]

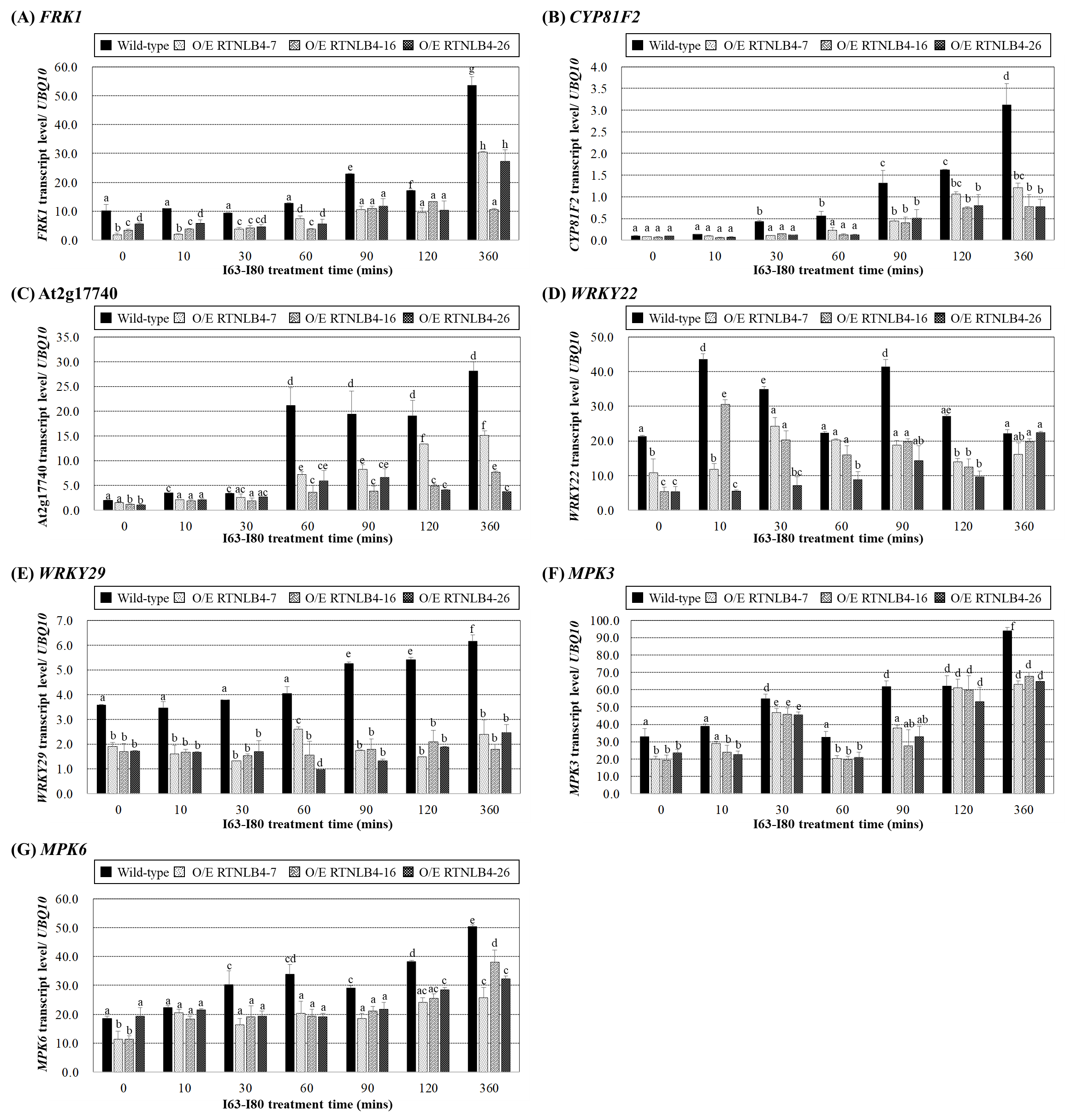


**Figure S4**. The VirB2 peptide, I63-I80, induced defense-related genes in wild-type plants. mRNA levels of *FRK1* (A), *CYP81F2* (B), At2g17740 (C), *WRKY22* (D), *WRKY29* (E), *MPK3* (F), and *MPK6* (G) in seedlings of wild-type and *RTNLB4* O/E transgenic plants elicited with 10 µM of I63-I80 peptide for 0, 10, 30, 60, 90, 120, and 360 min measured by qPCR analysis. *UBQ10* transcript level was an internal control. Data are mean±SE. Data were analyzed by Duncan tests and means with different letters were significantly different (P < 0.05).
