# Supplementary material for: Arabidopsis RETICULON-LIKE4 (RTNLB4) Protein Participates in Agrobacterium Infection and VirB2 Peptide-Induced Plant Defense Response
Source: Int J Mol Sci. 2020 Mar 3;21(5):1722. doi: 10.3390/ijms21051722 (PMC7084338; doi:10.3390/ijms21051722)
Supplement: Supplementary file 1 [file ijms-21-01722-s001.zip › Suppl figure and table/Figure S5-rtnlb4 mutant with S111T58-f.docx]

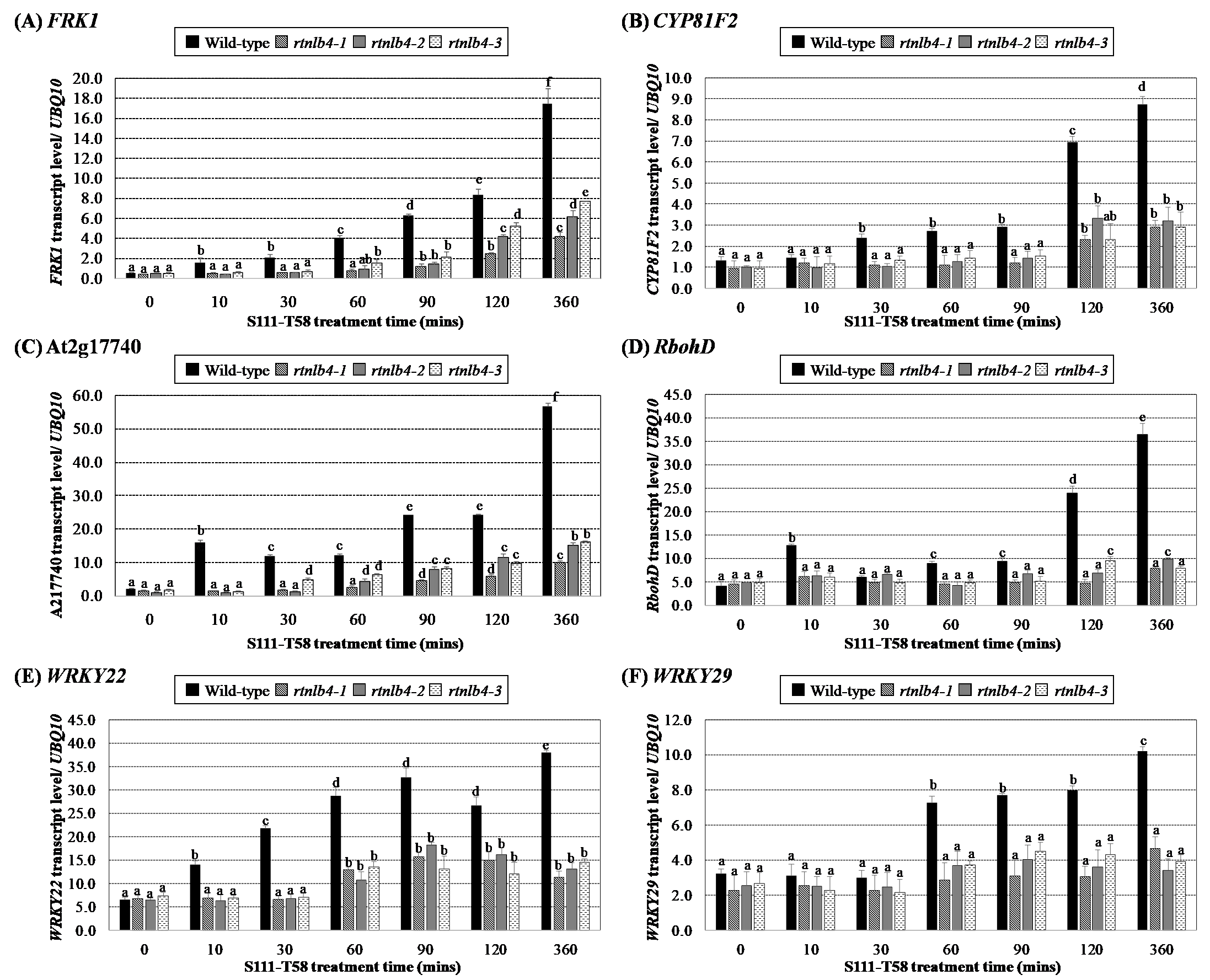


**Figure S5**. Induction of defense genes by VirB2 peptide, S111-T58, was decreased in three *rtnlb4* mutants. mRNA levels of *FRK1* (A), *CYP81F2* (B), At2g17740 (C), *RbohD* (D), *WRKY22* (E), and *WRKY29* (F) in seedlings of wild-type and three *rtnlb4* mutants elicited with 10 µM VirB2 peptide, S111-T58, for 0, 10, 30, 60, 90, 120, and 360 min measured by qPCR analysis. *UBQ10* transcript level was an internal control. Data are mean±SE. Duncan tests were used for statistical analysis and means with different letters were significantly different (P < 0.05).
