# Supplementary material for: Arabidopsis RETICULON-LIKE4 (RTNLB4) Protein Participates in Agrobacterium Infection and VirB2 Peptide-Induced Plant Defense Response
Source: Int J Mol Sci. 2020 Mar 3;21(5):1722. doi: 10.3390/ijms21051722 (PMC7084338; doi:10.3390/ijms21051722)
Supplement: Supplementary file 1 [file ijms-21-01722-s001.zip › Suppl figure and table/Figure S9-ROS assays control-f.docx]

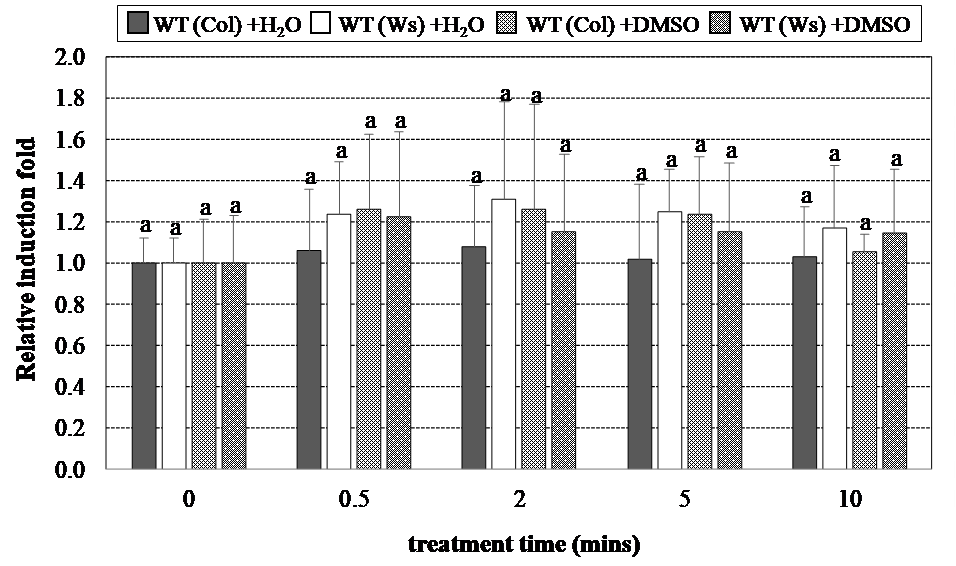


**Figure S9**. H_2_O_2_ amount in wild-type plants were not significantly different after the addition of dH_2_O or DMSO. H_2_O_2_ amount in wild-type plants was determined 0, 0.5, 2, 5, and 10 min after mock treatments. H_2_O_2_ amount at each time was normalized to the H_2_O_2_ amount at 0 min. Data are mean±SE from more than 10 plants. Data were analyzed by Duncan tests and means with same letters were not significantly different (P < 0.05).
