# Supplementary material for: Arabidopsis RETICULON-LIKE4 (RTNLB4) Protein Participates in Agrobacterium Infection and VirB2 Peptide-Induced Plant Defense Response
Source: Int J Mol Sci. 2020 Mar 3;21(5):1722. doi: 10.3390/ijms21051722 (PMC7084338; doi:10.3390/ijms21051722)
Supplement: Supplementary file 1 [file ijms-21-01722-s001.zip › Suppl figure and table/Supplementary Table 1-f.doc]

**Table S1. Primers and peptides used in this study.**

| **Primer names** | **Primer sequences** | **References** |
| --- | --- | --- |
| **Primers for generations of *RTNLB4* overexpression transgenic plants** | | |
| Forward primer of *RTNLB4*-5’-*Spe*I | 5’-GGACTAGTGGCGCGCCATGGTGGAAGACCACAAGCACGAG-3’ | This study |
| Reverse primer of *RTNLB4*-3’-*BamH*I | 5’-CGGGATCCATTTAAATTTAATCCTTCTTCTTGTTCAGAGC-3’ |  |
| **Primers for *rtnlb4* T-DNA insertion mutants** | | |
| Left T-DNA border primer LBb1.3 | 5’-ATTTTGCCGATTTCGGAAC-3’ | [79] |
| Left T-DNA border primer LBb1 | 5’-GCGTGGACCGCTTGCTGCAACT-3’ | [79] |
| Left T-DNA border primer LBc1 | 5’-AAACTGGAACAACACTCAACC-3’ | [79] |
| Forward primer of Km | 5’-ATGATTGAACAAGATGGATTG-3’ | [44] |
| Reverse primer of Km | 5’-TCAGAAGAACTCGTCAAGAA-3’ | [44] |
| Forward primer of 010299 | 5’-GTGAACAGGTTTCTCCCTTCC-3’ | This study |
| Reverse primer of 010299 | 5’-AGGACAATTCTCATCCACACG-3’ |  |
| Forward primer of 112109 | 5’-GAGCAAAAATCGCAAAGTGAC-3’ |  |
| Reverse primer of 112109 | 5’-GGATCCCACGGAAGAAGTTAG-3’ |  |
| Forward primer of 047050 | 5’-TATCTACCTCGATGCAAAGCG-3’ |  |
| Reverse primer of 047050 | 5’-CCCTCTTACGAGGACCAGAAC-3’ |  |
| **Primers for quantitative real-time PCR analysis** | | **References** |
| Forward primer of *RTNLB4* | 5’-ACTCGAGATATGGTGGAAGACCACAAGC-3’ | This study |
| Reverse primer of *RTNLB4* | 5’-TTCCATGGTTAATCCTTCTTCTTGTTCAG-3’ |  |
| Forward primer of *UBQ10* | 5’-CTGCGTCTTCGTGGTGGTTTCTA-3’ | This study |
| Reverse primer of *UBQ10* | 5’-GTCGAGTCACTTTGCAGGCGTATTA-3’ |  |
| Forward primer of *FRK1* | 5’-GCCAACGGAGACATTAGAG-3’ | [55] |
| Reverse primer of *FRK1* | 5’-CCATAACGACCTGACTCATC-3’ |  |
| Forward primer of *PR1* | 5’-GGAGCTACGCAGAACAACTAAGA-3’ | [85] |
| Reverse primer of *PR1* | 5’-ACGAGGATCATAGTTGCAACTGA-3’ |  |
| Forward primer of *WRKY22* | 5’-TCCTTCGGAGAGATTCGAGA-3’ | This study |
| Reverse primer of *WRKY22* | 5’-CTGCTGCTACATGGCACACT-3’ |  |
| Forward primer of *WRKY29* | 5’-TCCGGTACGTTTTCACCTTC-3’ | This study |
| Reverse primer of *WRKY29* | 5’-AGAGACCGAGCTTGTGAGGA-3’ |  |
| Forward primer of *MPK3* | 5’-TGACGTTTGACCCCAACAGA-3’ | This study |
| Reverse primer of *MPK3* | 5’-CTGTTCCTCATCCAGAGGCTG-3’ |  |
| Forward primer of *MPK6* | 5’-CCGACAGTGCATCCTTTAGCT-3’ | This study |
| Reverse primer of *MPK6* | 5’-TGGGCCAATGCGTCTAAAAC-3’ |  |
| Forward primer of *RbohD* | 5'-GATCAAGGTGGCTGTTTACCC-3' | [86] |
| Reverse primer of *RbohD* | 5'-TCGGCAGTTCACCAACATGA-3' | [86] |
| Forward primer of *CYP81F2* | 5'-CATACCACGTGGCACTATCG-3' | [87] |
| Reverse primer of *CYP81F2* | 5'-ACCAGGACACGTTCTTCGTC-3' | [87] |
| Forward primer of At2G17740 | 5’-TGCTCCATCTCTCTTTGTGC-3’ | [88] |
| Reverse primer of At2G17740 | 5’-ATGCGTTGCTGAAGAAGAGG-3’ | [88] |
| **Peptides for seedling treatments** | | |
| **Peptides names** | **Peptides sequences** | **References** |
| elf18 | MSKEKFERTKPHVNVGTI | [51] |
| Agro-flg22 | SRVSSGLRVKSASDNAAYWSIA | [24] |
| VirB2-S111-T58 | SFLGKTLTGGGQSAGGGTDPAT | This study |
| VirB2-I63-I80 | ICTFILGPFGQSLAVLGI |  |
| VirB2-I80V-101 | IVAIGISWMFGRASLGLVAGVV |  |
| VirB2-G95-F112 | GLVAGVVGGIVIMFGASF |  |
| VirB2-I104-G121 | IVIMFGASFLGKTLTGGG |  |

**References**

24.	Felix, G.; Duran, J.D.; Volko, S.; Boller, T. Plants have a sensitive perception system for the most conserved domain of bacterial flagellin. *Plant J.* **1999**, *18*, 265-276, 10.1046/j.1365-313x.1999.00265.x.

44.	Huang, F.C.; Fu, B.J.; Liu, Y.T.; Chang, Y.R.; Chi, S.F.; Chien, P.R.; Huang, S.C.; Hwang, H.H. *Arabidopsis* RETICULON-LIKE3 (RTNLB3) and RTNLB8 participate in *Agrobacterium*-mediated plant transformation. *Int. J. Mol. Sci.* **2018**, *19*, 638, 10.3390/ijms19020638.

51.	Kunze, G.; Zipfel, C.; Robatzek, S.; Niehaus, K.; Boller, T.; Felix, G. The N terminus of bacterial elongation factor Tu elicits innate immunity in Arabidopsis plants. *Plant Cell* **2004**, *16*, 3496-3507, 10.1105/tpc.104.026765.

55.	Po-Wen, C.; Singh, P.; Zimmerli, L. Priming of the *Arabidopsis* pattern-triggered immunity response upon infection by necrotrophic Pectobacterium carotovorum bacteria. *Mol. Plant Pathol*. **2013**, *14*, 58-70, 10.1111/j.1364-3703.2012.00827.x.

79.	Alonso, J.M.; Stepanova, A.N.; Leisse, T.J.; Kim, C.J.; Chen, H.; Shinn, P.; Stevenson, D.K.; Zimmerman, J.; Barajas, P.; Cheuk, R.; Gadrinab, C.; Heller, C.; Jeske, A.; Koesema, E.; Meyers, C.C.; Parker, H.; Prednis, L.; Ansari, Y.; Choy, N.; Deen, H.; Geralt, M.; Hazari, N.; Hom, E.; Karnes, M.; Mulholland, C.; Ndubaku, R.; Schmidt, I.; Guzman, P.; Aguilar-Henonin, L.; Schmid, M.; Weigel, D.; Carter, D.E.; Marchand, T.; Risseeuw, E.; Brogden, D.; Zeko, A.; Crosby, W.L.; Berry, C.C.; Ecker, J.R. Genome-wide insertional mutagenesis of *Arabidopsis thaliana*. *Science* **2003**, *301*, 653-657, 10.1126/science.1086391.

85.	Journot-Catalino, N.; Somssich, I.E.; Roby, D.; Kroj, T. The transcription factors WRKY11 and WRKY17 act as negative regulators of basal resistance in *Arabidopsis thaliana*. *Plant Cell* **2006**, *18*, 3289-3302, 10.1105/tpc.106.044149.

86.	Wang, Y.; Shen, W.; Chan, Z.; Wu, Y. Endogenous cytokinin overproduction modulates ROS homeostasis and decreases salt stress resistance in *Arabidopsis thaliana*. *Front. Plant Sci*. **2015**, *6*, 1004, 10.3389/fpls.2015.01004.

87.	Singh, P.; Chien, C.C.; Mishra, S.; Tsai, C.H.; Zimmerli, L. The Arabidopsis LECTIN RECEPTOR KINASE-VI.2 is a functional protein kinase and is dispensable for basal resistance to *Botrytis cinerea*. *Plant Signal. Bevhav*. **2013**, *8*, e22611, 10.4161/psb.22611.

88.	He, P.; Shan, L.; Lin, N.C.; Martin, G.B.; Kemmerling, B.; Nurnberger, T.; Sheen, J. Specific bacterial suppressors of MAMP signaling upstream of MAPKKK in *Arabidopsis* innate immunity. *Cell* **2006**, *125*, 563-575, 10.1016/j.cell.2006.02.047.
