# Supplementary material for: Arabidopsis RETICULON-LIKE4 (RTNLB4) Protein Participates in Agrobacterium Infection and VirB2 Peptide-Induced Plant Defense Response
Source: Int J Mol Sci. 2020 Mar 3;21(5):1722. doi: 10.3390/ijms21051722 (PMC7084338; doi:10.3390/ijms21051722)
Supplement: Supplementary file 1 [file ijms-21-01722-s001.zip › Suppl figure and table/Supplementary Table 2-f.doc]

**Table S2. Bacterial strains and plasmids used in this study.**

| **Strain/ plasmid** | **Relevant characteristics** | **Reference/ source** |
| --- | --- | --- |
| **Strains** |  |  |
| *A. tumefaciens* |  |  |
| GV3101(pMP90) | Rm^R^, Gm^R^, GV3101 harboring pTiC58ΔT-DNA | [77] |
| A208 | Rm^R^, A136 harboring wild type pTiT37, the bacteria for tumor assay | [85] |
| At849 (GV3101[pMP90] with pBISN1) | Rm^R^, Km^R^, GV3101 harboring pTiC58ΔT-DNA and a pBISN1 binary vector, the bacteria for transient transformation assay | [89] |
| C58C1(pTiB6S3ΔT) with pBISN1 | Rm^R^, Cb^R^, Tc^R^, Km^R^, C58C1 strain harboring pTiB6S3ΔT-DNA, a helper plasmid pCH32, and a binary vector pBISN1; the bacteria for the AGROBEST assay | [47,90] |
| C58 | Rm^R^, Wild-type virulent strain containing nopaline-type Ti plasmid pTiC58 | [91] |
| *E. coli* |  |  |
| DH10B | Host for DNA cloning | Invitrogen, Carlsbad, CA, USA |
| **Plasmids** |  |  |
| pBluescript II | Amp^R^, plasmid for the DNA cloning in *E. coli* | Agilent Technologies, Santa Clara, CA, USA |
| pBluescript-RTNLB4 | Amp^R^, a plasmid containing the coding sequence of *RTNLB4* from *Arabidopsis* | This study |
| pE1798 | Km^R^, a binary vector containing a double CaMV 35S promoter and a Nos terminator | [42] |
| pE1798-RTNLB4 | Km^R^, a binary vector containing a *RTNLB4* coding sequence driven by a double CaMV 35S promoter | This study |
| pET23a-RTNLB4 | Amp^R^, a plasmid containing the T7-tagged-RTNLB4 fusion protein | [42] |
| pE1798-T7-tag-RTNLB4 | Km^R^, a binary vector containing a T7-tagged-*RTNLB4* sequence driven by a double CaMV 35S promoter | This study |

**References**

42.	Hwang, H.H.; Gelvin, S.B. Plant proteins that interact with VirB2, the *Agrobacterium* pilin protein, mediate plant transformation. *Plant Cell* 2004, *16*, 3148-3167, 10.1105/tpc.104.026476.

47.	Wu, H.Y.; Liu, K.H.; Wang, Y.C.; Wu, J.F.; Chiu, W.L.; Chen, C.Y.; Wu, S.H.; Sheen, J.; Lai, E.M. AGROBEST: an efficient *Agrobacterium*-mediated transient expression method for versatile gene function analyses in *Arabidopsis* seedlings. *Plant Methods* **2014**, *10*, 19, 10.1186/1746-4811-10-19.

77.	Koncz, C.; Schell, J. The promoter of TL-DNA gene 5 controls the tissue-specific expression of chimaeric genes carried by a novel type of *Agrobacterium* binary vector. *Mol. Gen. Genet*. **1986**, *204*, 383-396, 10.1007/BF00269243.

85.	Journot-Catalino, N.; Somssich, I.E.; Roby, D.; Kroj, T. The transcription factors WRKY11 and WRKY17 act as negative regulators of basal resistance in *Arabidopsis thaliana*. *Plant Cell* **2006**, *18*, 3289-3302, 10.1105/tpc.106.044149.

89.	Nam, J.; Matthysse, A.G.; Gelvin, S.B. Differences in susceptibility of *Arabidopsis* ecotypes to crown gall disease may result from a deficiency in T-DNA integration. *Plant Cell* **1997**, *9*, 317-333, 10.1105/tpc.9.3.317.

90.	Deblaere, R.; Bytebier, B.; De Greve, H.; Deboeck, F.; Schell, J.; Van Montagu, M.; Leemans, J. Efficient octopine Ti plasmid-derived vectors for *Agrobacterium*-mediated gene transfer to plants. *Nucleic. Acids Res*. **1985**, *13*, 4777-4788, 10.1093/nar/13.13.4777.

91.	Lin, B.C.; Kado, C.I. Studies on *Agrobacterium tumefaciens* VIII avirulence induced by temperature and ethidium bromide. *Can. J. Microbiol*. **1977**, *23*, 1554-1561, 10.1139/m77-229.
